# Inconsistent descriptions of lumbar multifidus morphology: A scoping review

**DOI:** 10.1186/s12891-020-03257-7

**Published:** 2020-05-19

**Authors:** Anke Hofste, Remko Soer, Hermie J. Hermens, Heiko Wagner, Frits G. J. Oosterveld, André P. Wolff, Gerbrand J. Groen

**Affiliations:** 1 Anesthesiology Pain Center, University of Groningen, University Medical Center Groningen, Location Beatrixoord, Dilgtweg 5, Haren, the Netherlands; 2 Faculty of Physical Activity and Health, Saxion University of Applied Sciences, Enschede, the Netherlands; 3 Department of Biomedical Signals & Systems, Faculty of Electrical Engineering, Mathematics and Computer Science, University of Twente, Enschede, the Netherlands; 4 Telemedicine Group, Roessingh Research and Development, Enschede, the Netherlands; 5 Department of Movement Science, Institute of Sport and Exercise Sciences, Münster, Germany

**Keywords:** Paraspinal Muscles, Lumbar Vertebrae, Lumbar multifidus, Erector spinae, Magnetic Resonance Imaging, Ultrasonography, Computer Tomography, Scoping review, low back pain

## Abstract

**Background:**

Lumbar multifidus (LM) is regarded as the major stabilizing muscle of the spine. The effects of exercise therapy in low back pain (LBP) are attributed to this muscle. A current literature review is warranted, however, given the complexity of LM morphology and the inconsistency of anatomical descriptions in the literature.

**Methods:**

Scoping review of studies on LM morphology including major anatomy atlases. All relevant studies were searched in PubMed (Medline) and EMBASE until June 2019. Anatomy atlases were retrieved from multiple university libraries and online. All studies and atlases were screened for the following LM parameters: location, imaging methods, spine levels, muscle trajectory, muscle thickness, cross-sectional area, and diameter. The quality of the studies and atlases was also assessed using a five-item evaluation system.

**Results:**

In all, 303 studies and 19 anatomy atlases were included in this review. In most studies, LM morphology was determined by MRI, ultrasound imaging, or drawings – particularly for levels L4–S1. In 153 studies, LM is described as a superficial muscle only, in 72 studies as a deep muscle only, and in 35 studies as both superficial and deep. Anatomy atlases predominantly depict LM as a deep muscle covered by the erector spinae and thoracolumbar fascia. About 42% of the studies had high quality scores, with 39% having moderate scores and 19% having low scores. The quality of figures in anatomy atlases was ranked as high in one atlas, moderate in 15 atlases, and low in 3 atlases.

**Discussion:**

Anatomical studies of LM exhibit inconsistent findings, describing its location as superficial (50%), deep (25%), or both (12%). This is in sharp contrast to anatomy atlases, which depict LM predominantly as deep muscle. Within the limitations of the self-developed quality-assessment tool, high-quality scores were identified in a majority of studies (42%), but in only one anatomy atlas.

**Conclusions:**

We identified a lack of standardization in the depiction and description of LM morphology. This could affect the precise understanding of its role in background and therapy in LBP patients. Standardization of research methodology on LM morphology is recommended. Anatomy atlases should be updated on LM morphology.

## Background

Stabilizing therapy through muscle training is one of the main physiotherapeutic interventions for low back pain [1–3]. A uniform theoretical background for this treatment is lacking [4], however, and more recent studies report contradictory results following this treatment [5–7]. There are no explanations for how stabilizing therapy could have such opposing effects on patients with low back pain. Given that the lumbar multifidus (LM) is regarded as the major stabilizing muscle of the spine [5, 8–14], the anatomy and topography of this muscle might offer at least some explanation for the opposing effects of stabilizing therapy.

The morphology of the LM is complex, and several anatomical descriptions have appeared in the literature [15–19]. Anatomical studies have concluded that the LM has the largest cross-sectional area (CSA) of paraspinal muscles with short levers located at the most medial part of the spine between approximately L4 and S1 [16, 20, 21]. Important factors in spinal stabilization include CSA, deformation or stress in ligaments, and muscle type, activity pattern, force, mass, and length [8–10, 19, 22]. According to other studies, however, LM muscle mass is too small to play a substantial stabilizing role, and the primary stabilizing role should be attributed to the erector spinae (ES) [4]. Furthermore, an ongoing debate concerns the topography of the LM to the ES (i.e., deep vs superficial) [19, 23–25]. This discussion has led to many different approaches to investigating the morphology and functional characteristics of the LM. For example, LM muscle morphology has been quantified through ultrasound imaging (USI), MRI scanning, CT scanning, surgery, biopsy, and cadaver research. The outcomes of these methods have led to varying conclusions about CSA, muscle thickness, percentage of fat infiltration, fiber-bundle angle, and fiber length [19, 26–29]. Although each method has its own strengths and limitations [30], the results also depend on other variables, including population, spine-level measurement, and methodological quality.

At present, there is no clear overview of the similarities and differences between anatomy atlases and LM topography studies with regard to LM topography in humans. Such an overview is essential to improving understanding concerning the theoretical background of stabilizing therapies in the treatment of low back pain, as well as with regard to its role in basic anatomy training. The present study is therefore intended to review the literature on LM morphology.

## Materials and Methods

This study was conducted according to the guidelines formulated by Arksey and O’Malley 2005 and by Grudniewicz et al. 2016 [31, 32], using the following five-step framework: (1) identification of the research question, (2) identification of relevant literature, (3) study selection, (4) data extraction, and (5) collation, summary, and reporting of results. The identification of the research question (1) is explained in the Background section. Steps 2, 3, and 4 are explained in this Materials & Methods section, and Step 5 is discussed in the Results section.

### (2) Identification of relevant literature

#### Search strategy

To identify relevant studies on LM morphology, two databases—PubMed (Medline) and EMBASE—were searched, as well as gray literature (anatomy atlases) until June 2019. Search strategies were built, consisting of a combination of database-specific MeSH terms, title/abstract, free text, “wild cards” (words truncated by using “*”), and Boolean operators (“AND”, “OR”). The search string is provided in Additional file 1. The snowball method was used to identify additional papers from the reference lists of studies that were included.

#### Eligibility

All of the studies included were reviewed in terms of population, method, and outcome. To be included, studies had to be published in English and be based on studies of adult humans or human cadavers. A supplemental search of the Dutch literature did not reveal any relevant studies. Letters to the editor, abstract-only articles, and review papers were excluded. The initial search identified an extensive number of studies and gray literature. To minimize the inclusion of low-quality studies, we limited inclusion to peer-reviewed studies. All of the studies included were screened for the methods used to measure LM morphology: USI, MRI, CT scanning, modeling (biomechanical model of muscles), and cadaver studies. Furthermore, the parameters by which LM morphology was defined were described for each study (i.e., images, photos, drawings, models, trajectory descriptions, thickness or CSA, spine levels, and location of the LM).

### (3) Study selection

The selection procedure started with the identification of studies in the databases and the elimination of duplicates using the duplicate function in Endnote X9. Further, studies were screened according to title, abstract, and full-text, and additional papers were identified from reference lists of the included studies. Two authors (AH and RS) independently selected and assessed studies for quality and subsequently discussed them to reach consensus. When no consensus was achieved, a third reviewer was consulted (GJG).

Anatomy atlases were included as well, given their importance as basic anatomical introductions to LM topography. The anatomy atlases were selected through a university library system, followed by a snowball procedure, as they were not included in medical databases. Major anatomy atlases available in English, Dutch, and German were retrieved from the university libraries (including specialized medical libraries) of the University Medical Center Groningen and Saxion University of Applied Sciences, as well as from online resources. The results of anatomical studies and atlases are presented separately.

### (4) Data extraction

All studies and atlases were extracted according to the following LM parameters: location (deep/superficial), imaging methods, spine levels, muscle trajectory (origin and insertion), muscle diameter (anteroposterior diameter), and CSA. The risk of bias assessment was not determined, as it primarily has to do with the methodology of studies [33]. Instead, a quality-assessment tool was developed to rate the quality of the descriptions of LM morphology. The tool consists of five items, each worth one point, with a maximum score of five (Table 1). The reliability of LM morphology descriptions was assessed by checking for the presence of an image and determining whether this image was an original photograph (as opposed to a model or drawing) [34]. Furthermore, the validity of the images was assessed by checking for the labelling of the LM, depiction of spinal levels, and description of planes. Descriptions scoring 5/5 were regarded as being of high quality, with scores of (3-4)/5 representing moderate quality and scores (≤ 2)/5 representing low quality. The inter-rater reliability (% agreement) of the two reviewers was calculated using a kappa value. In cases where the LM location was not described explicitly despite the presence of adequate imaging, the LM location was determined in consensus by the authors (AH, RS and GG).

**Table 1:** Quality assessment tool

Item		Meaning of score
1	Image present	1 = yes; 0 = no
2	Quality of image	1 = sufficient (unambiguous for lumbar multifidus by MRI, photo, dissection, CT, ultrasound or biopsy) 0 = insufficient (tenuous for lumbar multifidus by modeling or drawing)
3	Clear labeling of LM	1 = yes; 0 = no
4	Presence of spine levels depicted	1 = yes; 0 = no
5	Description of plane	1 = yes; 0 = no

## Results

### Study selection

**Fig. 1:**
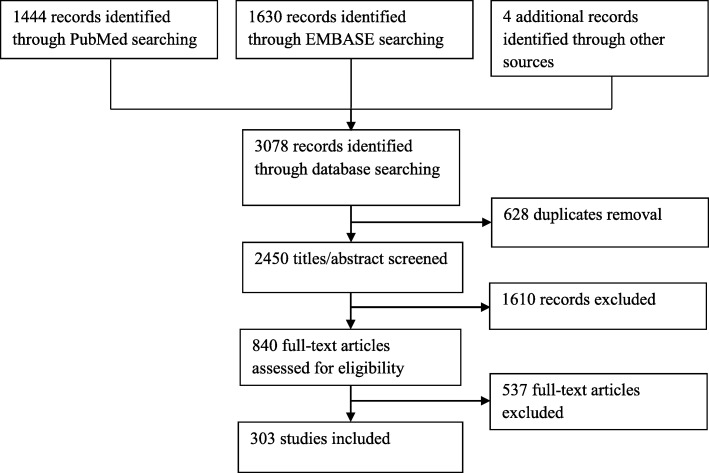
Flowchart of the selection process for the literature review

The search yielded 2450 original studies, 299 of which were ultimately included, along with 4 additional studies. In addition, 19 anatomy atlases were identified that described parameters of LM morphology (Additional file 2) [25, 55–67]. The study-selection procedure is depicted in Fig. 1.

### LM parameters in studies

The characteristics of the studies included are presented in Additional file 3 [17–20, 22, 26, 27, 29, 34–37, 39–53, 71–80, 82–343]. In descriptions of moderate to high quality, the most frequently applied methods for measuring or visualizing LM morphology were MRI (51%), USI (36%), and drawings (8%).

#### Location

In 153 of the 303 studies, LM was presented only as a superficial muscle at one or more levels between L4–S1. In 72 studies, it was presented only as a deep muscle and, in 35 studies, it was presented as both a superficial and a deep muscle. We were unable to identify the precise location of LM in 43 studies (Additional file 3).

#### Origin and insertion

The origin of the LM is described at the spinous process of L4 and L5 [35]. In some studies, however, LM origin was also described at the caudal and dorsal surface of each lamina (L1–L5) (Table 2) [23]. Whereas some studies described LM insertion as being at the lateral or medial side of the dorsal foramen of the sacrum [23, 35], others stated that the superficial LM muscle fibers are inserted at the posterior superior iliac spine (PSIS) [19, 23].

**Table 2:** Studies describing the fiber trajectory of LM

Author year	Method^1^	Spine Level	L^2^	Fiber trajectory of LM
[26] Beneck 2012	1	L4-S1	S	The LM morphology best captures span the L4-L5 or L5-S1 functional spinal units
[35] Bogduk 1992	6	L5-S1	U	LM origin is tip or shaft of spinous process L1-L5. LM insertion is medial and lateral next to the posterior sacral foramen
[36] Bojadsen 2000	4	T1-S1	D	LM insertion at the spinous process of L5 and of T12 and T11 contains vertical fibers. The most caudal fibers of LM run a vertical trajectory between the medial portion of the sacrum and the spinous process of L5.
[37] Creze 2017	2, 4	L3	D & S	On the first inspection, the multifidus represented a homogenous muscular mass with a triangular shape. It comprised many millimetric tendinous and fleshy fascicles originating from the spinous processes to the mammillary processes located 1–3 spinal levels above. The muscular organization was unclear and the multifidus appeared as a multiceps and multipennate muscle. Multifidus fascicles were arranged in three or four layers from superficial too deep with few or no cleavage planes between them. Some interdigitations attached fascicles between them. For each lumbar level, the muscular fascicles and fibers were oriented from 98 to 228 to the line of spinous processes.
[38] Creze 2018	4	-	S	The ES aponeurosis (ESA) had different anatomical relationships with the longissimus, the iliocostalis, and the LM. Along the lumbar and sacral regions, close to the SPL medially, some muscle fibers of superficial fascicles of the LM were attached directly (without pennation) to the ESA. Each fascicle of the LM (i.e., the group of muscles originating from a spinous process) was covered by a thin pearly white aponeurosis corresponding to a fascial expansion of the cranial attachment on the spinous process. Connectives fibers were all oriented longitudinally along the muscle belly. The thickness of the LM aponeurosis decreased along the rostrocaudal axis of each fascicle and was too thin to be measured with the material used. Connective fibers of the LM aponeurosis were oriented parallel to the longitudinal axis of the fascicle.
[39] Creze 2019	4	-	S	The cranial attachment was located on the spinous processes and caudal attachments on the mammillary processes of the three to four vertebras below, the sacrum and on the ESA. There was no tendon at the level of the sacrum, but there were aponeuroses as well as muscle fibers.
[40] De Foa 1989	4	L1-L2	D	LM fibers run parallel to a line between the posterior superior iliac spine and the L1-L2 interspinous space
[41] Jemmett 2004	4,5	L2-S1	D & S	The superficial LM fibers of the first fascicle of the LM originated at the caudolateral tip of the L1 spinous process. The deep LM fibers of the first fascicle originated from the caudolateral base of the L2 spinous process. This first fascicle inserted at the mamillary process and lamina of L4 as well as the capsule of the L4/5 zygapophysial joint and the most cranial aspect of the PSIS. The second fascicle originated in the same manner from the L2 andL3 spinous processes and inserted near the PSIS and just adjacent to the superior articular process of S1.
[42] Kader 2000	1	L3-S1	S	LM consists of five separate bands, each originating from a spinous process and spreading caudolaterally from the midline to be inserted into the mammillary processes of the facet joints, the iliac crest, and the sacrum. In an axial MR image the LM is displayed as two, three or four bands, depending on the level of the image
[18] Kim 2015	6	L1-L5	D	LM consists of laminar fibers, fascicles from the shaft and from the tip of the spinous process.
[43] Kramer 2001	1, 5	-	S	LM activity was measured with EMG at the level of the vertebral body of L2.
[44] Macintosh 1986	4, 5	L1-L5	D & S	The principal fascicles of the LM arise as a common tendon from the tubercle and from the lateral surface of the caudal edge of the spinous process. The caudal attachments of these fascicles are the mammillary processes, the iliac crest and dorsal surface of the sacrum
[19] Moseley 2002	2, 5	L4	D & S	LM EMG: The first electrode was inserted **±** 4 cm lateral to the midline and directed medially until it reached the lamina to make recordings from the LM fibers immediately adjacent to the lamina of L4, most likely those arising from the inferior edge of the L3 spinous process (i.e., deep multifidus). The second electrode was inserted **±** 4 cm from the midline and advanced to a depth of approximately 1 cm, medial to the lateral border of LM, to record the superficial LM fibers that arise from the upper lumbar vertebras. The third electrode was inserted **±** 2 cm lateral to the midline and advanced until it reached the spinous process **±** 1 cm from the superficial border of LM to record the superficial fibers of LM adjacent to the L4 spinous process
[45] Lonnemann 2008	4, 5	L1-S1	D & S	The superficial LM layer originated from the mamillary process to insert onto the tip of two spinous processes and supraspinous ligaments at the same vertebral level and one above. Tendinous slips and muscle tissue extended dorsally to the overlying ES aponeurosis. The second LM layer originated from the posteroinferior lateral aspect of the spinous processes as a common tendon. The third LM layer originated from the lateral aspect of the inferior aspect of the spinous processes as a muscular band of origin.
[29] Rosatelli 2008	4, 5	-	D & S	Superficial LM fibers originate via a common tendon from the tips of the spinous processes (L1–L5) and pass inferolateral to the mammillary processes of L5, S1, sacrum, and ilium. Intermediate LM fibers originate from the spinous processes of L1–L4. Distally, L1, L2, and L3 portions attach as tendons to the L4, L5, and S1 mammillary processes, respectively. However, the L4 portion attached to the sacrum at the S2 level. The deep LM contains five entirely muscular segments (L1–L5). Each segment attached superiorly to the lamina of L1–L5, and inferiorly two levels more caudal to the L3, L4, L5, and S1 mammillary process, respectively, while the L5 fascicle attached to the sacrum
[46] Vialle 2005	4	L4 - L5	S	An anatomical cleavage plane between LM and the longissimus part of the sacrospinalis muscle is present. The level of the natural cleavage plane between LM and longissimus was noted and measured between this level and the midline at the level of the spinous process of L4
[22] Ward 2009	4, 5	T12-S1	D & S	LM was identified by its position adjacent to the spinous process and the cranial/medial to caudal/lateral projection of its fibers. LM had isolated muscle bellies on the posterolateral region between L4 and S1.

^1^1 = MRI; 2 = USI; 3 = CT; 4 = Photo; 5 = Drawing; 6 = Modelling; 7 = Stereomicroscope; 8 = Tractography.

^2^L=Location; D = Deep; S = Superficial

#### Muscle thickness and cross-sectional area (CSA)

**Fig. 2:**
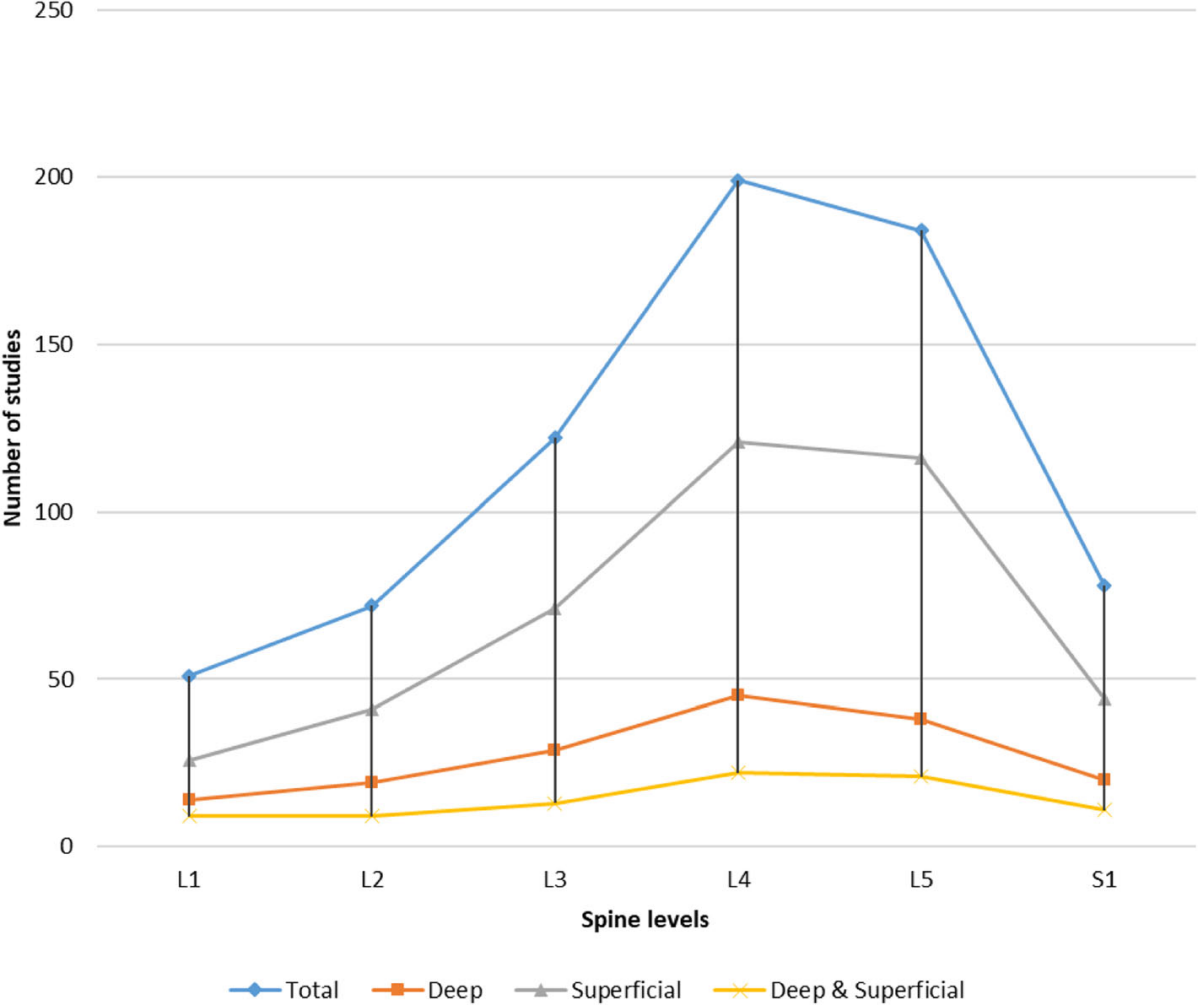
Overview of spine levels at which LM is measured in all studies (Total), in studies of moderate to high quality referring to deep LM (Deep), in studies referring to superficial LM (Superficial), and in studies referring to deep and superficial LM (Deep & Superficial). Deep muscles lie closer to bone or internal organs, and superficial muscles are close to the surface of the skin

We identified a variety of methods of measuring the CSA of LM. These methods include USI, CT scanning, and/or MRI at various levels of the lumbar spine (Additional file 3) between L1 and S1 (Fig. 2). This focus on L4 and L5 measurements was found in nearly all studies on different locations (Fig. 2).

The CSA of LM has been measured in a variety of populations, resulting in an extensive range of LM CSA outcomes [27, 47, 48]. The total range in square millimeters varied between 9.08 and 2500 mm^2^, possibly due to the incorrect description of corresponding units of value. Variations in LM thickness were found with regard to the level of measurement (L3/L4, L4/L5, or L5/S1) and LM activation conditions (rest vs (sub)maximal voluntary contraction), as well as in terms of body position (e.g., prone vs standing position) (Additional file 4) [38, 47–53, 51–53, 74, 75, 98, 108, 113, 115, 127, 131, 135, 136, 144, 151, 159, 162, 163, 168, 175, 187, 203, 211–213, 233, 235, 236, 239, 253, 259, 263, 286, 301, 305, 308, 309, 311, 317–322, 325, 326, 335, 336, 340] [48–53]. The total range in LM thickness in millimeters varied between 2.4 and 41.1 mm [53, 54].

### LM parameters in anatomy atlases

Within the anatomy atlases, we observed variations in the description and presentation of LM (Table 5), although the majority of atlases showed the same configuration of the LM. In 16 of the 19 atlases reviewed, the LM was depicted as a deep back muscle [24, 25, 55–62, 64–71], either covered by the thoracolumbar fascia and/or as being covered by the ES. Moreover, LM imaging varied in terms of the presence of spine levels (cervical-sacrum), imaging planes (transversal, dorsal, sagittal), and of whether it was with or without other low back muscles in a single figure.

#### Location and muscle diameter

Variations were found with regard to the location, diameter, and topography of the LM. In one anatomy atlas (*Gray’s Anatomy*) [61], the superficial part of the LM extended from T11 cranially to S3 caudally as a wide (large anterior-posterior diameter) muscle next to the median sacral crest. In a *Radiology Anatomy Atlas Viewer* [63], the LM was depicted in the axial spinal cross-sections, albeit with inconsistent labelling of the LM.

Variations were found in the diameter of the LM between the various lumbar levels. The location of the widest part of the LM varied between the level of PSIS [55, 58, 60, 61] and L5–S1 [25, 56, 57, 69]. In some atlases, however, the widest part of the LM was undefined, due to the overlying low back muscles [24, 67].

Various origins and insertions of the LM were identified in the anatomy atlases (Table 3), with the (lumbar) multifidus extending between the dorsal part of the sacrum [69] and the transverse processes of T1 [24, 25, 55–58, 60, 65, 67, 70, 71], and as attaching to the iliac [68] or ischium [55, 60] part of the pelvis.

**Table 3:** Atlases describing the fiber trajectory of lumbar multifidus

Author year	Method^2^	Spine Level	L^1^	Fiber trajectory	
				Origin	Insertion
[56] A.M. Gilroy 2014	1	C2-S5	D	C2-sacrum: transverse and spinous processes with crossing to 2 to 4 vertebra	
[57] M. Schuenke 2010	1	C2-sacrum	D	Courses between the transversus and spinous processes (2-4 vertebras) or all cervical vertebras most fully developed in the lumbar spine.	
[58] K.L. Moore 2010	1	C4 - sacrum	D	Origin: LM arises from the posterior sacrum, PSIS of the ilium, aponeurosis of erector spinae, sacroiliac ligaments mammillary processes of lumbar vertebras, transverse processes of T1-T3, articular processes of C4-C7 distal attachment: thickest in the lumbar region. fibers pass obliquely super medially to the entire length of spinous processes, located 2-4 segments, superior to the proximal attachment.	
[60] K.L. Moore 2011	1	C4-sacrum	D	LM origin: arises from the posterior sacrum, PSIS of the ilium, aponeurosis of erector spinae, sacroiliac ligaments, mammillary processes of lumbar vertebras, transverse processes of T1-T3 and articular processes of C4-C7	LM insertion: Thickest in the lumbar region, fibers pass obliquely superomedially to the entire length of spinous processes of vertebras located 2-4 segments superior to the origin.
[70] W. Dauber 2006	1	C1-sacrum	D	LM: sacrum, processes mammillary of vertebras. Thoracic multifidus: transverse processes. Cervical multifidus: caudal processes of cervical vertebras.	LM: spinous processes L5-L1. Thoracic multifidus: spinous processes T12-T1. Cervical multifidus: spinous processes C7-C2.
[65] R.L. Drake 2010	1	T1-sacrum	D	Sacrum, the origin of erector spinae, PSIS, mammillary processes of lumbar vertebras, transverse processes of thoracic vertebras, and articular processes of lower four cervical vertebras	The base of spinous processes of all vertebras from L5 to C2 (axis)
[61] S. Standring 2008	1	C2-sacrum	D	At each segmental level multifidus is formed by several fascicles that arise from the caudal side of the lateral surface of the spinous process and from the caudal side of its tip. They radiate caudally to insert into the transverse elements of vertebrae two, three, four and five levels below (Machintosh et al. 1986). These sites are represented at lumbar levels by the mammillary processes. Fascicles that extend beyond the fifth lumbar vertebra insert into the dorsal surface of the sacrum. The longest fascicles from the first and second lumbar vertebras insert into the dorsal segment of the iliac cest. From each spinous process, the shortest fascicles pass inferiorly and laterally to their insertion; the longer fascicles assume a progressively steeper course and are arranged progressively more medially. These fascicles from a given segment are flanked and overlapped dorsolateral by fascicles from successively higher segments, an arrangement that endows the intact muscle with a laminated structure.	
[25] M. Schunke 2010	1	C2-sacrum	D	Origin and insertion: multifidus run between transverses processes and spinous processes (across 2 to 4 vertebras) of the whole spine (C2 to the sacrum). LM is strongest developed in the lumbar spine.	
[67] W. Platzer 2012	1, 2	C2-sacrum	D	M. multifidus runs from the sacrum to C2. The muscle fibers arise separately from the superficial tendon of m. longissimus of the dorsal plan of sacrum, transverse processes of thoracic vertebras and the articular processes of C2-C7.	

^1^ L=Location; D = Deep

^2^ 1 = MRI; 2 = USI

Overall, deep LM trajectories were consistently described between L1 and S5 [25, 56–58, 60, 61, 67, 70], although some superficial LM fibers were illustrated as originating from the spinous process of T10 [69] or T12 [61, 70]. Furthermore, some atlases did not illustrate the origin and insertion of the LM, as other muscles were more superficially presented and/or because these features were not described [24, 59, 62, 64, 66, 68].

### Quality assessment

Quality scores were determined for each description and anatomical image of the LM in the literature. The per-item quality scores for descriptions and anatomical images are presented in Table 4. The agreement between the reviewers of the quality assessment had a kappa value of 0.67, and all differences were resolved in a consensus meeting. There were no major differences between the descriptions and anatomical images of the LM in the literature with regard to the presence of images. The most difficult item to score was clear labeling.

**Table 4:** Total scores for quality items, in numbers and percentages

*N* (%)	Studies (*n*=303)	Atlases (*n*=19)
Image present (yes)	252 (83%)	19 (100%)
Quality of images (sufficient)	238 (79%)	6 (32%)
Clear labeling (yes)	200 (66%)	19 (100%)
Presence of spine levels depicted (yes)	236 (78%)	10 (53%)
Description of plane (yes)	232 (77%)	10 (53%)

**Fig. 3:**
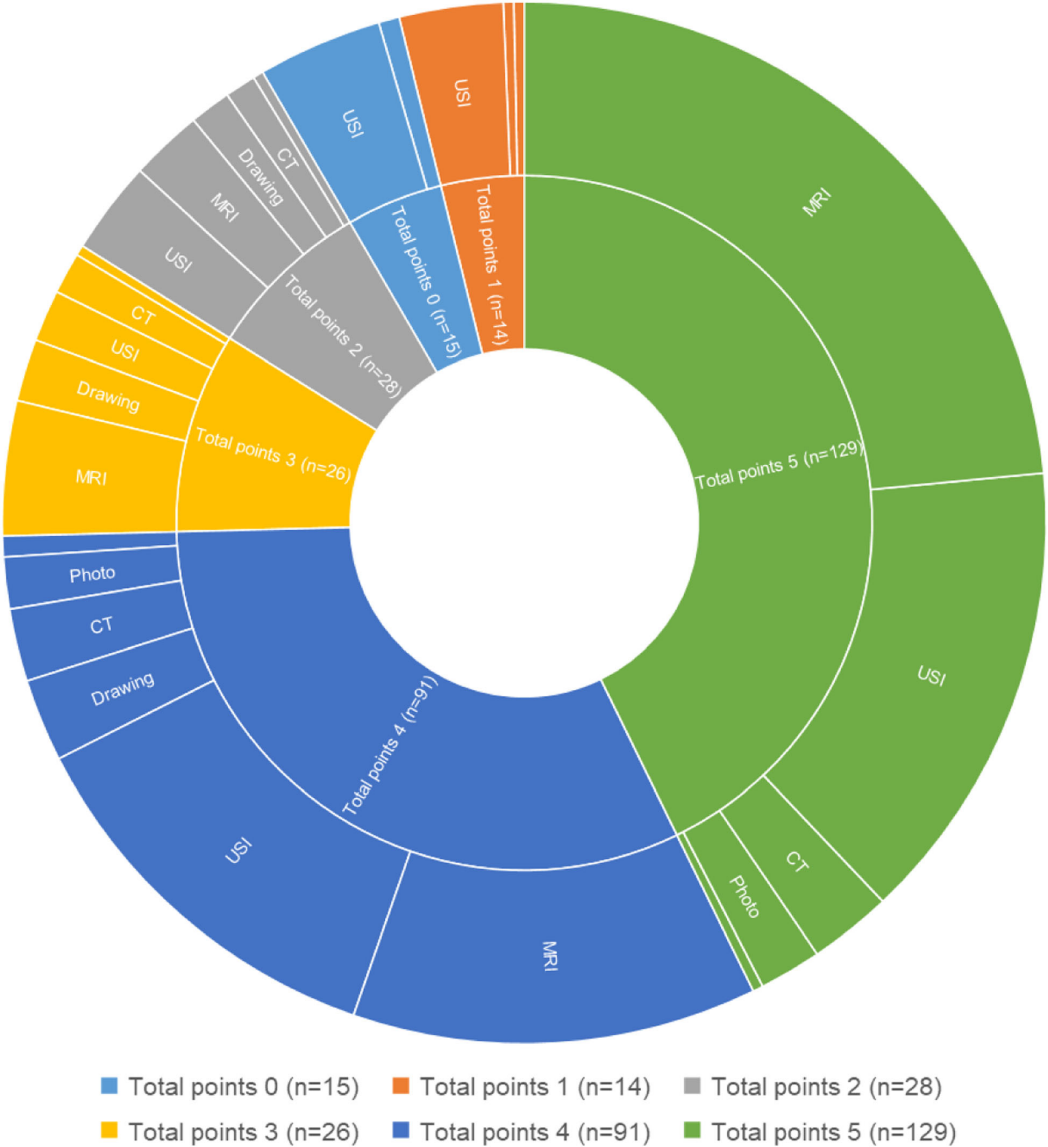
Overview of the percentage of total scores on the quality-assessment tool (inside ring) and the associated percentage of techniques used (outside ring). Scores per study are presented in Additional file 3

The total quality scores of the studies varied between 1 and 5 (out of 5). The highest score [5/5] was found in 43% of the studies (129/303), with moderate scores [[2–4]/5] found in 39% of the studies (117/303) and low scores [(≤2)/5] found in 19% of the studies (57/303). In the studies with quality scores of 5/5 and 4/5, MRI and USI were the most commonly used methods for visualizing the LM muscle (Fig. 3). More detailed data are presented in Additional file 3. The majority of atlases were rated as being of moderate quality [(3–4)/5] (79%, 15/19) or low quality [(≤2)/5] (16%, 3/19). Only one atlas was found to be of high quality [5/5] (Table 5) [55].

**Table 5:** Data extraction of the atlases included (n=19), sorted by quality score

Author year	Method^2^	Spine Level	L^1^	Quality ^3^
[55] L.G.F. Giles 1997	3, 4	L1-sacrum	D	5
[56] A.M. Gilroy 2014	3	C2-S5	D	4
[57] M. Schuenke 2010	3	C2-sacrum	D	4
[58] K.L. Moore 2010	3	C4 - sacrum	D	4
[59] J.W. Rohen 2011	4, 2	L1 and L4	D	4
[60] K.L. Moore 2011	3	C4-sacrum	D	4
[61] S. Standring 2008	3	C2-sacrum	D	4
[62] T.B. Moeller 2007	3, 2	L5	D	4
[63] R. Livingston 2011	1	Abdomen, Pelvis	D	4
[64] P.H. Abrahams 2013	4	T12-sacrum	D	3
[65] R.L. Drake 2010	3	T1-sacrum	D	3
[25] M. Schunke 2010	3	C2-sacrum	D	3
[66] G.Y. El-khoury 2007	1, 2	T10-sacrum	D	3
[67] W. Platzer 2012	3, 5	C2-sacrum	D	3
[24] F. Paulsen 2011	3	C2-sacrum	D	3
[68] P. Kopf-Maier 2000	3	C2-sacrum	D	3
[69] P.W. Tank 2009	3	T1-sacrum	D	2
[70] W. Dauber 2006	3	C1-sacrum	D	2
[71] H.J. van Donkelaar 2007	3, 5	C2-sacrum	D	2

^1^ L=Location; D = Deep

^2^ 1 = MRI; 2 = USI; 3 = CT; 4 = Photo; 5 = Drawing

^3^ 5 = high quality; 4 or 3 = moderate quality; ≤ 2 = low quality

## Discussion

Substantial contradictory results were found across a large number of anatomy studies included in the review, and there appears to be no general consensus concerning the trajectory and muscle description of the LM [19, 23, 25, 29, 35]. Particularly with regard to the descriptions of “fiber trajectory” and “location”, major differences were found between the studies by Macintosh and Bogduk (1986), Rosatelli et al. (2008), and Moseley et al. (2002), and those by Kim et al. (2015), Bojadsen et al. (2000), and De Foa et al. (1989) [17–19, 29, 36, 40]. Discrepancies were also identified with regard to LM diameter, especially the distance between the spinous process and the lateral margin of the LM at levels L4–S1 [46, 72] and its location relative to the ES [46, 72, 73].

Each method that is used in literature to measure LM characteristics has its own strengths and limitations [30]. The architecture and function of the LM has been studied predominantly according to morphological and imaging methods. One disadvantage of cadaver studies [15, 17, 22, 29, 36, 40, 46, 74] is that the studies do not clearly identify the type and amount of structures (skin, fat, fascia, and muscle) that were removed from the cadaver. In these studies, it could be difficult to describe the exact location of the LM relative to other lumbar muscles and structures. The MRI and USI methods offer the advantage of being able to present undisturbed anatomy. This finding could have positive implications for clinical practice, given that USI is a user-friendly and affordable way to measure LM morphology in physiotherapy practice.

In anatomy atlases, the LM was depicted primarily as a relatively small deep muscle, in contrast to some research studies that refer to its large size and the presence of superficial slips at L4–S2 levels. Differences in LM images were identified even within anatomy atlases [25, 60, 61, 68]. In *Wolf-Heidegger’s Atlas of Human Anatomy*, LM insertion is depicted at the ventral side of the sacrum, in contrast to *Gray’s Anatomy*, in which it is depicted at the dorsal side of the sacrum [61, 68]. Furthermore, in some atlases, the diameter and location of the LM is undefined, due to the overlying low back muscles [24, 67]. Overall, anatomy atlases reflect no consensus about the fiber trajectory of the LM, thus making it difficult for therapists, clinicians, and students to know and learn what is correct about LM morphology.

The inconsistencies in the descriptions and imaging of LM morphology in studies and atlases could be due to the differences in many parameters. Some of these variables are related to methodology (Fig. 2), spine levels, and/or type of population [26, 36, 75, 76]. Of particular note are the variations we found between LM images in anatomy atlases and those in studies. In some large studies [15, 26, 77, 78], the location and presentation of the LM differ from those in the most recent anatomy atlases [24, 25, 55]. One consistent finding in the anatomy atlases was that they all depict the LM as a deep lumbar muscle, whereas most studies presented it as a superficial lumbar muscle at the levels of L4–S1. We nevertheless identified some consistency in studies based on a USI LM protocol that had been developed in an earlier study (e.g., by Belavy et al.) [79–81]. The same protocol, which referred to similar images, has been used in different studies based on different research questions to present new knowledge about LM morphology.

### Limitations

One possible limitation of this study could be that it might have overlooked some anatomy atlases, due to the lack of a database of anatomy atlases. Another limitation could be related to the reliability and validity of the quality-assessment tool that was developed and used by three authors of the current study. This quality-assessment tool was developed for lack of an existing “risk-of-bias assessment tool” with which to assess the quality of descriptions of LM morphology. It would be advisable to improve this assessment tool by conducting a validation and/or reliability study, as well as by expanding the tool beyond the current five items [34]. Any validation study regarding this quality-assessment tool will nevertheless be hampered by the current lack of a gold standard.

Variations in the images and measurement of LM morphology could be influenced by a number of potentially confounding factors, including research methods, level, side of measurement, population, intra-individual differences, intervention, research objectives, measurement technique (e.g., with or without contraction), and the relative experience of the assessors and/or practitioners creating the images. The variation that we observed in LM morphology emphasizes the importance of correct reference to morphology, although no gold standard for LM morphology has been developed to date. To reduce some of the existing variation, the authors call for improvement in the standardization of research protocols (e.g., in studies using EMG, USI, or MRI). The proper measurement of LM function could allow measurement of the contribution of the LM to spine movements in patients with non-specific LBP or other conditions. This knowledge could help clinicians and therapists to improve their diagnosis of patients.

### Clinical implications

Remarkable differences in the reporting of LM morphology were found within anatomical studies, as well as between anatomical studies and anatomy atlases, especially with regard to trajectories of the musculature and its location relative to the ES. Such differences in the reporting of LM could have implications for clinical practice, given that knowledge of morphology provides the foundation for the diagnosis and treatment of patients by physiotherapists. For example, if the topography of a low back muscle (in terms of origin, insertion, deep/superficial) is clear, it should provide a clearer indication of the function of this low back muscle. This could make it easier to identify a cause or diagnosis of low back function or muscle impairment. For therapists or clinicians, these inconsistencies make it difficult to conclude which results are correct. Once therapists or clinicians know the correct LM morphology, this will clarify the function of the LM. Such knowledge could enhance understanding concerning the role of the LM in patients with LBP. It could also enhance the quality and consistency of decision-making by specialists concerning treatments for patients with LBP. Although a recent review on the effects of stabilizing therapy compared to usual care identified significant benefits of stabilizing therapy on pain and disability, these differences were not interpreted as clinically important [82]. Improved diagnosis may allow better sub-grouping, possibly enhancing the therapeutic effects for patients with LBP.

## Conclusion

We identified a lack of standardization in the depiction and description of LM morphology, which may affect the precise understanding of its role in the background and therapy for patients with LBP. Standardization of research methodology with regard to LM morphology is recommended. Anatomy atlases should be updated on LM morphology.

## Supplementary information

**Additional file 1.** Search string.

**Additional file 2.** Included anatomy atlases.

**Additional file 3.** Data extraction of all included studies.

**Additional file 4.** Studies that measured thickness of lumbar multifidus.

**Additional file 5.** Legend of Additional files 3 and 4.

## Data Availability

Not applicable.

## Abbreviations

CSA: Cross-sectional area
ES: Erector spinae
LM: Lumbar multifidus
PSIS: Posterior superior iliac spine
USI: Ultrasound imaging
