# Supplementary material for: Inconsistent descriptions of lumbar multifidus morphology: A scoping review
Source: BMC Musculoskelet Disord. 2020 May 19;21:312. doi: 10.1186/s12891-020-03257-7 (PMC7236939; doi:10.1186/s12891-020-03257-7)
Supplement: Supplementary file 1 — Additional file 1. Search string. [file 12891_2020_3257_MOESM1_ESM.docx]

**Additional file 1**

*PubMed*
("Paraspinal Muscles"[Mesh] OR multifid*[tiab] OR semispinali*[tiab] OR interspinale*[tiab] OR intertransversa*[tiab] OR splenius[tiab] OR deep muscle*[tiab] OR intrinsic muscle*[tiab] OR paraspinal muscle*[tiab] OR rotatore*[tiab])
AND
("Lumbar Vertebrae"[Mesh] OR "Lumbosacral Region"[Mesh] OR "Low Back Pain"[Mesh] OR ((low[tiab] OR lower[tiab]) AND (back pain*[tiab] OR backache*[tiab] OR back ache*[tiab])) OR lumbago[tiab] OR low back[tiab] OR lumbar[tiab] OR lumbosacral[tiab] OR trunk[tiab] OR abdominal[tiab] OR ((spine[tiab] OR spinal[tiab]) AND stabil*[tiab]))
AND
("diagnostic imaging" [Subheading] OR "Magnetic Resonance Imaging"[Mesh] OR "Tomography, X-Ray Computed"[Mesh] OR "Ultrasonography"[Mesh] OR "Action Potentials"[Mesh] OR "Cadaver"[Mesh] OR OR "Computer Simulation"[Mesh:NoExp] OR computational[tiab] OR electromyogra*[tiab] OR cross-sectional area*[tiab] OR cross-section area*[tiab] OR MRI[tiab] OR magnetic resonance[tiab] OR imaging[tiab] OR ct-scan*[tiab] OR computed tomograph*[tiab] OR echograph*[tiab] OR ultraso*[tiab] OR modeling[tiab] OR modelling[tiab] OR thickness[tiab] OR size[tiab] OR fiber[tiab] OR fibre[tiab] OR morpholog*[tiab] OR sonoanatom*[tiab] OR anatomy[tiab] OR anatomic*[tiab] OR action potential*[tiab] OR cadaver*[tiab])
NOT
(("Animals"[Mesh] NOT "Humans"[Mesh]) OR "Review" [Publication Type])

*EMBASE*('multifidus muscle'/exp OR 'paraspinal muscle'/exp OR 'back muscle'/mj OR (multifid* OR semispinali* OR interspinale* OR intertransversa* OR splenius OR ‘deep muscle*‘ OR ‘intrinsic muscle* ‘ OR ‘paraspinal muscle*‘ OR rotatore*):ab,ti)
AND
('lumbar spine'/exp OR 'low back pain'/exp OR 'lumbosacral region'/exp OR ((low OR lower) NEXT/5 (back OR backache*)):ab,ti OR (lumbago OR lumbar OR lumbosacral OR trunk OR abdominal):ab,ti OR ((spine OR spinal) NEAR/5 stabil*):ab,ti)
AND
('radiodiagnosis'/exp OR 'echography'/exp OR 'cross sectional area'/exp OR 'action potential'/exp OR 'cadaver'/de OR 'computational model'/exp OR 'computational analysis'/exp OR 'computer simulation'/exp OR (‘cross-sectional area*‘ OR ‘cross-section area*‘ OR MRI OR ‘magnetic resonance‘ OR imaging OR ‘ct-scan*‘ OR ‘computed tomograph*‘ OR echograph* OR ultraso* OR modeling OR modelling OR computational:ab,ti OR thickness OR size OR fiber OR fibre OR morpholog* OR sonoanatom* OR anatomy OR anatomic* OR ‘action potential*‘ OR cadaver*):ab,ti)
NOT
(('animal'/exp NOT 'human'/exp) OR 'conference abstract'/it)
