# Supplementary material for: Inconsistent descriptions of lumbar multifidus morphology: A scoping review
Source: BMC Musculoskelet Disord. 2020 May 19;21:312. doi: 10.1186/s12891-020-03257-7 (PMC7236939; doi:10.1186/s12891-020-03257-7)
Supplement: Supplementary file 2 — Additional file 2. Included anatomy atlases. [file 12891_2020_3257_MOESM2_ESM.docx]

**Additional file 2**

| Included anatomy atlases. | | | |
| --- | --- | --- | --- |
| Title | Author(s) | Publisher | Year (edition) |
| (55) Anatomical atlas (In Dutch: Anatomische atlas) | A.M. Gilroy, B.R. MacPherson, L.M. Ross, M. Schunke, E. Schulte, U. Schumacher | Bohn Stafleu van Loghum | 2014 (2) |
| (56) Atlas of Anatomy: General Anatomy and Musculoskeletal System | M. Schuenke, E. Schulte, U. Schumacher | Thieme | 2010 (1) |
| (58) Lippincott Williams & Wilkins: Atlas of Anatomy | P.W. Tank, T.R. Gest, W. Burkel | Wolters Kluwer | 2009 (Latin) |
| (57) McMinn and Abrahams’ Clinical atlas of human anatomy | P.H. Abrahams, J.D. Spratt, M. Loukas, A. Van Schoor | Elsvevier | 2013 (7) |
| (59) Clinically Oriented Anatomy | K.L. Moore, A.F. Dalley, A.M.R. Agur | Wolters Kluwer | 2010 (6) |
| (61) Colour atlas of Anatomy | J.W. Rohen, C. Yokochi, E.Lutjen-Drecoll | Wolters Kluwer | 2011 (7) |
| (60) Essential Clinical Anatomy | K.L. Moore, A.M. R. Agur, A.F. Dalley | Wolters Kluwer | 2011 (4) |
| (62) Feneis’ illustrated anatomical pocket-dictionary (In Dutch: Feneis' Geillustreerd anatomisch zakwoordenboek) | W. Dauber(original author Heinz Feneis) | Bohn Stafleu van Loghum | 2006 (4) |
| (63) Gray's Anatomy for Students | R.L. Drake, A.W. Vogl, A.W.M. Mitchell | Elsevier | 2010 (2) |
| (64) Gray's Anatomy | S. Standring | Elsevier | 2008 (14) |
| (65) Clincal anatomy and embryology part 2 (In Dutch: Klinische anatomie en embryologie deel 2) | H.J. van Donkelaar, A.H.M. Lohman, A.F.M. Moorman | Elsevier | 2007 (3) |
| (66) Pocket Atlas of Sectional Anatomy: Computed Tomography and Magnetic Resonance Imaging, Volume II | T.B. Moeller, E. Reif | Thieme | 2007 (3) |
| (25) Anatomical atlas Prometheus: General anatoy and musculoskeletal system (In Dutch: Anatomische atlas Prometheus: Algemene anatomie en bewegingsapparaat) | M. Schunke, E. Schulte, U. Schumacher | Bohn Stafleu van Loghum | 2010 (2) |
| (67) Sectional anatomy by MRI and CT | G.Y. El-khoury, W.J. Montgomery, R.A. Bergman | Elsevier | 2007 (3) |
| (68) SESAM Atlas of the musculoskeletal system anatomy part 1. (In Dutch: SESAM Atlas van de anatomie Bewegingsapparaat deel 1) | W. Platzer | Thieme | 2012 (22) |
| (24) Sobotta: atlas of the human anatomy part 1. General anatomy of musculoskeletal system. (In Dutch: Sobotta: Atlas van de menselijke anatomie Deel 1. Algemene anatomie en bewegingsapparaat) | F. Paulsen, J. Waschke | Bohn Stafleu van Loghum | 2011 (4) |
| (69) Wolf-Heidegger's Atlas of Human Anatomy | P. Kopf-Maier | Karger | 2000 (5) |
| (54) Clinical anatomy and management of low back pain. | L.G.F. Giles LGF, K.P. Singer | Butterworth Heinemann | 1997 |
| (70) Radiology Anatomy Atlas Viewer | R. Livingston | Bearboat SP | 2011 |
