# Supplementary material for: Inconsistent descriptions of lumbar multifidus morphology: A scoping review
Source: BMC Musculoskelet Disord. 2020 May 19;21:312. doi: 10.1186/s12891-020-03257-7 (PMC7236939; doi:10.1186/s12891-020-03257-7)
Supplement: Supplementary file 3 — Additional file 3. Data extraction of all included studies. [file 12891_2020_3257_MOESM3_ESM.docx]

**Additional file 3**

| Data extraction of all included studies (n=303), sorted by quality score. | | | | | |
| --- | --- | --- | --- | --- | --- |
| Studies |  |  |  |  |  |
| Author year | Method^1^ | Spine  Level | L^1^ | Quality^2^ | CSA (mm^2^)^3^ |
| (82) Abbas 2016 | 3 | L3 | S | 5 |  |
| (83) Altinkaya 2016 | 1 | L3-S1 | S | 5 | 778.4^A,C^; 719.9^A,D^; 666.4^A,E^; 779.7^B,C^; 742.8^B,D^; 736.1^B,E^ |
| (84) Annaswamy 2013 | 1 | L1-L5 | S | 5 | 240.852^A,E,G,H^; 218.389^A,E,F,H^; 295.133^A,E,G,I^; 274.200^A,E,F,I^; 415.250^A,E,G,J^; 419.950^A,E,F,J^; 626.267^A,E,G,K^; 606.983^A,E,F,K^; 815.167^A,E,G,L^; 798.852^A,E,F,L^ |
| (85) Bang 2018 | 1 | L4-L5 | S | 5 |  |
| (86) Banno 2017 | 3 | L4-L5 | S | 5 | 565^A,P,MM^; 780^A,P,U,MM^ |
| (78) Belavy 2011 | 1 | L1-L5 | S | 5 | 240^B,H,T,U^; 290^B,I,T,U^; 400^B,J,T,U^; 520^B,K,T,U^; 650^B,L,T,U^ |
| (79) Belavy 2015 | 2, 1 | L1-L5 | S | 5 | 250^B,H^; 367^B,I^; 559^B,J^; 829^B,K^; 970^B,L^, 260^B,H^; 351^B,I^; 493^B,J^; 732^B,K^; 912^B,L^ |
| (80) Belavy 2017 | 2 | L1-L5 | S | 5 | 640^B,T,U^; 540^B,T,V^; 590^B,T,V^ |
| (26) Beneck 2012 | 1 | L4-S1 | S | 5 |  |
| (87) Berry 2018 | 1 | L4 | S | 5 | 9.08^A,E,F,K^; 9.14^A,E,G,K^ |
| (35) Bojadsen 2000 | 4 | T1-S1 | D | 5 |  |
| (88) Bresnahan 2017 | 1 | L4-L5 | S | 5 |  |
| (89) Cawley 2014 | 2 | T12-L5 | U | 5 |  |
| (90) Chang 2016 | 1 | L3-L4 | U | 5 |  |
| (91) Chatani 2016 | 1 | - | D / S | 5 |  |
| (92) Chon 2017 | 1 | L4-S1 | S | 5 | 635.6^A,D,P^; 659.4^B,D,P^; 698.5^A,D,Q^; 727.0^B,D,Q^; 619.6^A,E,P^; 666.5^B,E,P^; 708.4^A,E,Q^; 733.5^B,E,Q^ |
| (93) Clark 2009 | 1 | L3-L4 | U | 5 |  |
| (94) Coldron 2003 | 2 | L5 | S | 5 | 548^B,G,L,CCC^; 554^B,F,L,CCC^ |
| (95) Creze 2018 | 1 | L3-L5 | D | 5 |  |
| (96) Cuellar 2016 | 2 | L2-S1 | S | 5 | 293^I,G^; 290^I,F^; 378^J,G^; 386^J,F^; 474^K,G^; 484^K,F^; 531^L,G^; 539^L,F^ |
| (97) Danneels 2001 | 1 | L3-L4 | S | 5 | 0.341^E,J,R,DD^; 0.547^E,K,R,DD^; 0.71^E,K,S,DD^ |
| (98) Deng 2015 | 4, 3, 1, 5 | L1-S1 | S | 5 |  |
| (99) D'Hooge 2012 | 1 | L3-L4 | S | 5 | 41.0^E,K,R^; 37.5^I,K,R^; 40.9^E,B,K,R^; 41.0^E,A,K,R^ |
| (100) D'Hooge 2013 | 1 | L4 | S | 5 |  |
| (101) Dickx 2010 | 1 | L4 | D / S | 5 |  |
| (102) Dickx 2008 | 1 | L3-L5 | D | 5 |  |
| (103) Dickx 2010 | 1 | L3-L4 | D | 5 |  |
| (47) Djordjevic 2015 | 2 | L1-L5 | S | 5 |  |
| (104) Fan 2010 | 1 | L3-S1 | S | 5 | 1162.4^A,E,T,Z,AA^; 734.6^A,E,Z,AA^; 1058.6^A,E,T,Z,BB^; 929.8^A,E,Z,BB^ |
| (105) Farshad 2014 | 1 | L4-S1 | S | 5 |  |
| (48) Fortin 2015 | 1 | L3-S1 | S | 5 | 721^E,O^; 1224^E,Q^ |
| (106) Gellhorn 2017 | 1 | L5 | S | 5 | 1007^A,L^ |
| (107) Ghiasi 2016 | 1 | L1-S1 | S | 5 | 28.4^E,Q,CC^; 20.8^E,P,CC^; 14.5^E,O,CC^; 15.6^E,N,CC^; 22.2^E,CC,LL^ |
| (108) Gibbon 2017 | 2 | L4-L5 | D | 5 |  |
| (109) Gildea 2013 | 1 | L2-L5 | S | 5 |  |
| (110) Hides 1992 | 1, 2 | L2-S1 | S | 5 | 196^B,I,W,OO^; 329^B,J,W,OO^; 499^B,K,W,OO^; 715^B,L,W,OO^ |
| (111) Hides 2008 | 2 | L2-L5 | D | 5 | 279^B,I^; 434^B,J^; 653^B,K^; 804^B,L^ |
| (112) Hides 2011 | 1 | L3-L5 | S | 5 | 599^B,J,T^; 957^B,K,T^; 1148^B,L,T^ |
| (113) Hides 2014 | 2 | L5 | S | 5 | 914^B,P,T^ |
| (114) Hides 2016 | 2 | L5 | S | 5 |  |
| (115) Hides 2016 | 2 | L2-S1 | S | 5 | 263^B,I^; 449^B,J^; 674^B,K^; 784^B,L^ |
| (116) Hides 2017 | 2 | L4-L5 | S | 5 | 920^B,P^ |
| (117) Hiepe 2014 | 1, 6 | L2-L4 | S | 5 |  |
| (118) Hiepe 2015 | 1 | L3-L4 | S | 5 | 739^B,G,O,OO^; 785^B,F,O,OO^; 639^B,G,O,PP^; 648^B,F,O,PP^ |
| (119) Hyun 2007 | 1 | L3-S1 | S | 5 | 546.11^A,E,O^; 711.80^A,E,P^; 761.25^A,E,Q^ |
| (40) Jemmett 2004 | 4, 5 | L2-S1 | D / S | 5 |  |
| (120) Jiang 2017 | 1 | L4-L5 | S | 5 | 1585.0^A,P^; 1668.5^B,P^ |
| (121) Johnson 2002 | 6 | L3 | U | 5 |  |
| (122) Kader 2008 | 5, 1 | L4-L5 | D | 5 |  |
| (123) Kamaz 2007 | 3 | L1-L5 | S | 5 | 307^A,K,R,W^; 380^B,K,R,W^; 459^A,K,S,W^; 565^B,K,S,W^ |
| (124) Kang 2007 | 1 | L3-L5 | S | 5 | 1313.93^A,E,P^ |
| (125) Kang 2013 | 1, 5 | L4-S1 | S | 5 | 894.80^A,E,K,AAA^; 1017.14^A,E,L,AAA^; 855.07^A,E,K,AAA^; 1005.09^A,E,L,AAA^ |
| (126) Katsu 2018 | 1 | L3 | D | 5 |  |
| (75) Kiesel 2007 | 2 | L4-S1 | D | 5 |  |
| (127) Kiesel 2007 | 2 | L4-L5 | S | 5 |  |
| (128) Kiesel 2008 | 2 | L4-L5 | D / S | 5 |  |
| (76) Kim 2011 | 1 | L4-L5 | S | 5 | 680.1^A,C,P^; 664.7^B,C,P^; 632.9^A,E,P^; 675.3^B,E,P^ |
| (129) Koppenhaver 2012 | 2 | L4-L5 | S | 5 |  |
| (130) Kulig 2009 | 1, 5 | L4-L5 | S | 5 | 15.8^E,L,KK^ |
| (53) Lariviere 2013 | 2 | L2-L5 | S | 5 |  |
| (131) Lariviere 2018 | 2 | L3-S1 | S | 5 |  |
| (132) Lee 2014 | 1 | L3, L5 | S | 5 | 710^A,E,G,J,LL^; 680^A,E,F,J,LL^; 1100^A,E,G,L,LL^; 1090^A,E,F,L,LL^; 590^A,E,G,J,MM^; 580^A,E,F,J,MM^; 1000^A,E,G,L,MM^; 1020^A,E,F,L,MM^ |
| (133) Lee 2017 | 3 | L1-S1 | S | 5 |  |
| (134) Liu 2013 | 2, 5 | L1-S1 | D / S | 5 |  |
| (44) Lonnemann 2008 | 4, 5 | L1-S1 | D / S | 5 |  |
| (135) MacDonald 2011 | 2 | L4-S1 | D / S | 5 |  |
| (17) Macintosh 1986 | 4, 5 | L1-S1 | D / S | 5 |  |
| (136) Masaki 2015 | 2 | L4 | D | 5 |  |
| (137) Massé-Alarie 2016 | 2 | L5 | D / S | 5 |  |
| (138) Oh 2008 | 1, 7 | L2-S1 | S | 5 | 711.36^E,J^; 744.03^E,K^; 953.84^E,L^ |
| (139) Paalanne 2011 | 1 | L3-L4 | S | 5 | 793^B,G,K,R,LL^; 799^B,F,K,R,LL^; 583^B,G,K,R,MM^; 582^B,F,K,R,MM^ |
| (71) Palmer 2011 | 1 | L1-S1 | S | 5 |  |
| (140) Pressler 2006 | 5, 2 | S1 | D | 5 |  |
| (141) Ranson 2006 | 1 | L1-S1 | D | 5 |  |
| (142) Ranson 2008 | 1 | L1-S1 | D | 5 | 380^B,H^; 450^B,I^; 750^B,J^; 1010^B,K^; 1120^B,L,R^; 1170^B,L,S^ |
| (29) Rosatelli 2008 | 4, 5 | L1-S1 | D / S | 5 |  |
| (143) Sakai 2017 | 1 | L4-L5 | D | 5 |  |
| (144) Sions 2015 | 2 | L4-L5 | S | 5 |  |
| (145) Sions 2017 | 2, 1 | L4 | D | 5 |  |
| (146) Sitilertpisan 2011 | 2 | L2-L5 | S | 5 | 289^A,I,W,QQ,SS^; 288^A,I,W,RR,SS^; 430^A,J,W,QQ,SS^; 432^A,J,W,RR,SS^; 772^A,K,W,QQ,SS^; 775^A,K,W,RR,SS^; 958^A,L,W,QQ,SS^; 957^A,L,W,RR,SS^ |
| (147) Smuck 2015 | 1 | L1-S1 | S | 5 | 620^A,E,P,Q,T^ |
| (148) Stokes 2005 | 2 | L4-L5 | S | 5 | 787^B,K,LL^; 555^B,K,MM^; 891^B,L,LL^; 665^B,L,MM^ |
| (149) Storheim 2017 | 1 | L3-S1 | D / S | 5 |  |
| (150) Suh 2017 | 1 | L4-L5 | S | 5 |  |
| (151) Sweeney 2014 | 2 | L4-S1 | S | 5 |  |
| (152) Tabaraee 2015 | 1 | L2-S1 | D | 5 | 794.0^E,P,R,QQ^; 787.9^E,P,R,RR^ |
| (153) Teichtahl 2015 | 1 | L1-S1 | S | 5 | 700^A/B,O^ |
| (154) Teichtahl et al 2015 | 1 | L3-L4 | D | 5 | 690 ^A/B,O,UU^; 690 ^A/B,O,VV^; 720 ^A/B,O,WW^ |
| (155) Teyhen 2012 | 2 | L4-L5 | S | 5 |  |
| (156) Urrutia 2018 | 1 | L1-S1 | D | 5 |  |
| (157) Van 2006 | 2 | L4-L5 | S | 5 |  |
| (158) Wallwork 2007 | 2 | L2-L5 | S | 5 |  |
| (159) Wallwork 2009 | 2 | L2-L5 | S | 5 | 194^B,I^; 309^B,J^; 461^B,K^; 556^B,L^; 240^A,I^; 302^A,J^; 347^A,K^; 381^A,L^ |
| (160) Wan 2015 | 1 | L3-L4 | D | 5 | 645^A,E,R,SS^; 464^A,E,SS^; 381^A,E,S,SS^; 683^B,E,R,SS^; 533^B,E,SS^; 427^B,E,S,SS^ |
| (161) Watanabe 2018 | 1 | L4 | D | 5 |  |
| (162) Wilson 2016 | 2 | L1-S1 | S | 5 | 259^B,I,PP^; 333^B,J,PP^; 441^B,K,PP^; 504^B,L,PP^ |
| (163) Worsley 2012 | 2 | L3-L4 | S | 5 | 690^O,EEE^; 687^O,FFF^ |
| (164) Yagi 2016 | 1 | L5-S1 | D | 5 | 779.8^A,E,Q^; 488.7^A,E,Q^ |
| (165) Yanik 2013 | 1 | L4-L5 | S | 5 |  |
| (77) Yoo 2014 | 3 | L3-S1 | S | 5 | 1224.0^B^; 610.9^B,SS^ |
| (49) Zapata 2015 | 2 | L1, L4 | S | 5 |  |
| (166) Zielinski 2013 | 2 | L2-L5 | S | 5 |  |
| (167) Zotti 2017 | 1 | L4-L5 | S | 5 |  |
| (168) Azadinia 2019 | 2 | L4 | D | 5 |  |
| (169) Banno 2019 | 1 | L1,L5 | S | 5 | 278^E,M,PP^; 636^E,P,PP^ |
| (170) Belavy 2019 | 1 | L4 | S | 5 | 217^B,H,OO^; 305^B,I,OO^; 450^B,J,OO^; 638^B,K,OO^; 782^B,L,OO^; |
| (171) Berry 2019 | 1 | L4 | S | 5 |  |
| (172) Burkhart 2019 | 3 | L2 | D | 5 |  |
| (173) Crawford 2019 | 1 | L1,L4 | D / S | 5 |  |
| (174) Emami 2018 | 2 | L4 | D | 5 |  |
| (175) Fortin 2019 | 2 | L5-S1 | D / S | 5 | 8.96^G,Q,W,MM,CCC^; 9.01^F,Q,W,MM,CCC^; 10.38^G,Q,W,MM,DDD^; 10.48^F,Q,W,MM,DDD^ |
| (176) Hanimoglu 2019 | 1 | L4,L5 | S | 5 | 16.12^A,P,W,^ |
| (177) Huang 2019 | 1 | - | D | 5 |  |
| (178) Lindstrom 2019 | 3 | L1-L5 | S | 5 |  |
| (179) Liu 2019 | 1, 5 | L1-S1 | S | 5 |  |
| (180) Mannil 2018 | 1 | L3,L4 | D | 5 |  |
| (181) Menezes-Reis 2018 | 1 | L4,L5 | S | 5 | 12.91^B,G,O,W^; 12.3 ^B,F,O,W^; 17.6^B,G,P,W^; 17,4^B,F,P,W^; 18.1^B,G,Q,W^; 17.1^B,F,Q,W^ |
| (182) Minetto 2018 | 2 | L5 | S | 5 |  |
| (183) Quittner 2018 | 1 | L1-L5 | S | 5 | 441.6^B,W^; 518.4^B,W,LL^; 380.6^B,W,MM^ |
| (184) Rezazadeh 2019 | 1 | L4-S1 | S | 5 |  |
| (185) Sarafraz 2019 | 2 | L5 | S | 5 | 51.39^A,L,X,CCC^; 46.54^B,L,X,CCC^ |
| (186) Sadeghi 2019 | 2 | L4,L5 | D / S | 5 |  |
| (187) Shadani 2019 | 1 | L2-L5 | D | 5 |  |
| (188) Sions 2019 | 2 | L4,L5 | S | 5 |  |
| (189) Smyers Evanson 2018 | 2 | L1-L5 | D | 5 | 730^B,G,L,W,MM,CCC^;791^B,G,L,W,LL,CCC^; 738^A,G,L,W,MM,CCC^; 777^A,G,L,W,LL,CCC^ |
| (190) Takashima 2018 | 1 | L4,L5 | S | 5 | 520.5^A,G,P,^ |
| (191) Wagner 2018 | 1 | L3,L4 | D | 5 | 526.0^A,O^; 499.1^A,E,O^ |
| (192) Wesselink 2019 | 1 | L4,L5 | S | 5 |  |
|  |  |  |  |  |  |
| (193) Alejaldre 2012 | 1, 3 | - | S | 4 |  |
| (194) Arocho-Quinones 2018 | 1 | L5-S1 | S | 4 |  |
| (195) Atci 2016 | 1 | L4-L5 | S | 4 |  |
| (196) Baek 2014 | 2 | L4-L5 | D / S | 4 |  |
| (197) Baek 2014 | 2 | L4-L5 | D / S | 4 |  |
| (198) Baek 2017 | 2 | L4-L5 | D / S | 4 |  |
| (199) Battaglia 2014 | 1 | L4-S1 | D | 4 |  |
| (20) Battie 2012 | 1 | L3-S1 | D | 4 | 910^A,P^; 920^B,P^; 1020^A,Q^; 960^B,Q^ |
| (200) Belavy 2008 | 1 | L1-S1 | S | 4 | 840^T,U^; 850^T,V^ |
| (201) Belavy 2010 | 1 | L1-S1 | S | 4 | 245.5^B,H,U^; 330.2^B,I,U^; 472.2^B,J,U^; 753.1^B,K,U^; 952.1^B,L,U^; 278.3^B,H,V^; 378.7^B,I,V^; 531.6^B,J,V^; 716.5^B,K,V^; 878.4^B,L,V^ |
| (202) Belavy 2011 | 1 | L1-S1 | S | 4 | 245.5^B,H^; 330.2^B,I^; 472.2^B,0^; 753.1^B,K^; 952.1^B,L^ |
| (26) Beneck 2013 | 2 | L4 | D / S | 4 |  |
| (34) Bogduk 1992 | 6 | L5-S1 | U | 4 |  |
| (203) Brenner 2007 | 2 | L4-S1 | S | 4 |  |
| (204) Campbell 1998 | 2, 1 | L5 | D / S | 4 |  |
| (205) Chen 2014 | 1 | L5 | S | 4 |  |
| (206) Chen 2018 | 3 | L4-S1 | D | 4 |  |
| (207) Chung 2013 | 3 | L2-L5 | D | 4 | 355.37^A,E,I,T^; 586.03^A,E,J,T^; 919.75^A,E,K,T^; 1187.14^A,E,L,T^ |
| (36) Creze 2017 | 4, 2 | L3 | D / S | 4 |  |
| (208) Danneels 2000 | 3 | L3-L4 | S | 4 | 0.47^B,J,R,YY^; 0.41^E,J,R,YY^; 0.63^B,K,R,YY^; 0.58^E,K,R,YY^; 0.9^B,K,S,YY^; 0.77^E,K,S,YY^ |
| (209) Danneels 2016 | 1 | L3-L5 | S | 4 |  |
| (39) De Foa 1989 | 4 | L1-L2 | D | 4 |  |
| (210) De Ridder 2015 | 1 | L4 | S | 4 |  |
| (211) Debuse 2013 | 2 | L4-L5 | D | 4 |  |
| (212) Dickx 2010 | 2 | L3-S1 | D | 4 |  |
| (213) Djordjevic 2014 | 2 | L4-L5 | D | 4 |  |
| (214) Dreyfuss 2009 | 1 | L4-S1 | U | 4 |  |
| (215) Ekin 2016 | 1 | L4-S1 | S | 4 |  |
| (216) Fischer 2013 | 1 | L4-L5 | U | 4 |  |
| (217) Fortin 2014 | 1 | L3-S1 | S | 4 | 721^E,O,T^; 709^E,O^; 1224^E,Q,T^; 1094^E,Q^ |
| (218) Fortin 2017 | 1 | L5 | S | 4 |  |
| (219) Goubert 2017 | 1 | L3-L4 | S | 4 |  |
| (220) Hides 2017 | 2 | L5 | S | 4 | 753^B,L,T^; 935^B,L^ |
| (221) Hides 1995 | 1, 2 | L2-S1 | S | 4 | 202^B,I,MM^; 333^B,J,MM^; 487^B,K,MM^; 712^B,L,MM^, 196^B,I,MM^; 329^B,J,MM^; 499^B,K,MM^; 715^B,L,MM^ |
| (46) Hides 2008 | 2 | L4 - L5 | S | 4 | 244^A,E,I,W,SS^; 334 ^A,E,J,W,SS^; 340 ^A,E,K,W,SS^; 322 ^A,E,L,W,SS^ |
| (222) Hides 2011 | 1 | L1-L5 | S | 4 | 260.1^B,H,T^; 347.8^B,I,T^; 487.0^B,J,T^; 730.3^B,K,T^; 938.2^B,L,T^ |
| (223) Hussein 2016 | 1 | L3-S1 | D | 4 |  |
| (41) Kader 2000 | 1 | L3-S1 | S | 4 |  |
| (224) Kanchiku 2014 | 5 | L4 | S | 4 |  |
| (18) Kim 2015 | 6 | L1-L5 | D | 4 |  |
| (225) Kjaer 2007 | 1 | L3-S1 | S | 4 |  |
| (74) Koppenhaver 2009 | 2 | L4-L5 | S | 4 |  |
| (226) Koppenhaver 2009 | 2 | L4-L5 | S | 4 |  |
| (227) Koppenhaver 2011 | 2 | L4-S1 | D | 4 |  |
| (228) Larrie-Baghal 2012 | 2 | L2-L5 | S | 4 | 320^B,G,I,MM^; 319^B,F,I,MM^; 398^B,G,J,MM^; 395^B,F,J,MM^; 490^B,G,K,MM^; 490^B,F,K,MM^; 587^B,G,L,MM^; 566^B,F,L,MM^ |
| (229) Lee 2006 | 2 | L4-L5 | S | 4 | 762^B,F,K^; 714^B,F,L^; 768^B,G,K^; 725^B,G,L^; 695^A,F,K^; 700^A,F,^L; 720A^,G,K^; 695^A,G,L^ |
| (72) Li 2016 | 1 | L1 - S1 | S | 4 |  |
| (43) Macintosh 1986 | 5 | L1-L5 | D / S | 4 |  |
| (230) Mayer 2015 | 2 | L4-L5 | S | 4 | 1120^B,G,K^; 1200^B,F,K^; 1130^B,G,L^; 1120^B,F,L^; 1100^B,F,K^; 1130^B,F,K^; 1110^B,G,L^; 1100^B,F,K^ |
| (231) Mangum 2016 | 2 | L5 | S | 4 |  |
| (232) McKiernan 2015 | 2 | - | S | 4 |  |
| (233) Nabavi 2014 | 2 | L4-L5 | S | 4 | 4290^G,P^; 4380^F,P^ |
| (234) Nuzzo 2013 | 2 | L4-S1 | S | 4 |  |
| (235) Nuzzo 2014 | 2 | L4-L5 | S | 4 | 1160^B,K,LL^; 1150^B,L,LL^; 760^B,K,MM^; 900^B,L,MM^ |
| (236) Partner 2014 | 2 | L4-L5 | D / S | 4 |  |
| (73) Rantanen 1994 | 5 | L1-S1 | D / S | 4 |  |
| (237) Scott 2015 | 2 | L5 | S | 4 | 870^A,F,L^; 880^A,G,L^; 810^B,F,L^; 850^B,G,L^ |
| (238) Sebro 2016 | 3 | L3-L5 | U | 4 |  |
| (239) Seung 2007 | 3, 5 | - | D | 4 | 1121.3^E,T^; 1122.9^E,T^ |
| (240) Shafaq 2012 | 1 | L1-S1 | S | 4 | 24.9^A,M,NN^; 35.2^A,N,NN^; 52.4^A,O,NN^; 77.0^A,P,NN^; 91.8^A,Q,NN^ |
| (241) Shahidi 2017 | 1 | L4 | S | 4 | 929^E,K,MM^; 1149^E,K,LL^ |
| (242) Shahtahmassebi 2017 | 2 | L4-S1 | S | 4 |  |
| (51) Sions 2014 | 2, 5 | L3-S1 | S | 4 |  |
| (189) Smyers 2018 | 2 | L1-L5 | D | 4 | 205^B,F,H,MM^; 207^E,F,H,MM^; 271^B,F,H, LL^; 277^E,F,H, LL^; 203^B,G,H,MM^; 190^E,G,H,MM^; 261^B,G,H,LL^;252^E,G,H,LL^ |
| (243) Stokes 1992 | 5 | L4 | S | 4 |  |
| (244) Tonomura 2017 | 1 | - | D / S | 4 |  |
| (245) Urrutia 2018 | 1 | L1-S1 | D | 4 |  |
| (246) Valentin 2015 | 1 | L1-L5 | D | 4 |  |
| (247) Valentin 2015 | 1 | L1-L5 | D / S | 4 |  |
| (45) Vialle 2005 | 4 | L4-S1 | S | 4 |  |
| (248) Watson 2008 | 2 | L5 | D | 4 |  |
| (249) Willemink 2012 | 1 | L1-S1 | D | 4 |  |
| (250) Winslow 2017 | 1 | L4-S1 | S | 4 |  |
| (251) Wong 2013 | 2 | L3 | S | 4 |  |
| (252) Woodham 2014 | 5, 1 | L3-S1 | D / S | 4 |  |
| (253) Aboufazeli 2019 | 2 | L4,L5 | D | 4 |  |
| (254) Agten 2018 | 2, 4 | L4 | S | 4 |  |
| (37) Creze 2018 | 4 | - | S | 4 |  |
| (255) Creze 2019 | 4 | - | S | 4 | 24^B,PP^ |
| (256) Izumoto 2019 | 1 | L1,L3, L5 | S | 4 |  |
| (257) Lorbergs 2018 | 3 | L3 | D | 4 |  |
| (258) Murillo 2019 | 2 | L3 | D / S | 4 |  |
| (259) Pishnamaz 2018 | 2 | L3-L5 | S | 4 |  |
| (187) Shadani 2018 | 2 | L5 | U | 4 |  |
| (260) Xiao 2018 | 1 | L4-S1 | S | 4 |  |
| (261) Xie 2019 | 1 | L1-S1 | D | 4 |  |
| (262) Yaltirik 2018 | 1 | L4-S1 | S | 4 | 558^A,D^; 722^A,D^ |
| (263) Yoshiko 2018 | 2 | L4,L5 | U | 4 |  |
| (264) Yurdakul 2019 | 2 | L4 | S | 4 |  |
| (265) Zhang 2018 | 1 | L3 | U | 4 |  |
| (266) Zhu 2018 | 1, 5 | - | D / S | 4 |  |
|  |  |  |  |  |  |
| (267) Akgul 2013 | 1 | L2-L5 | S | 3 | 368.91^A,G,I,YY^; 355.67^A,F,I,YY^; 529.69^A,G,J,YY^; 511.29^A,F,J,YY^; 693.15^A,G,K,YY^; 697.51^A,F,K,YY^; 762.08^A,G,L,YY^;759.63^A,F,L,YY^ |
| (268) Anaya 2014 | 1 | L3, L5 | S | 3 |  |
| (269) Barker 2004 | 1 | L2-S1 | D | 3 | 49.7^A,G^; 59.5^B,^F; 61.7^B,G^; 49.7^A,F^ |
| (270) Bouche 2011 | 3 | L3-L5 | S | 3 | 1325^A,J,ZZ,BBB^; 1095^A,J,AAA,BBB^; 1722^A,K,ZZ,BBB^; 1438^A,K,AAA,BBB^; 1949^A,L,ZZ,BBB^; 1556^A,L,AAA,BBB^ |
| (271) Cai 2015 | 2 | - | S | 3 |  |
| (272) Fan 2010 | 1 | L3-S1 | S | 3 | 1211.98^E,T,AA^; 703.95^E,AA^; 1066.69^E,T,BB^; 975.24^E,BB^ |
| (273) Gala-Alarcon 2018 | 2 | L4-L5 | S | 3 | 3870^B,P,X^; 129^B,P,X^ |
| (274) Gilbert 2018 | 1 | L1-L5 | S | 3 |  |
| (275) Hadar 1983 | 3 | L1-S1 | D | 3 |  |
| (276) Han 1992 | 5 | L3-L4 | U | 3 |  |
| (277) Hides 2012 | 1 | L2-L5 | S | 3 | 360^B,I^; 620^B,J^; 940^B,K^; 108^B,L^ |
| (278) Hildebrandt 2017 | 1 | L3-L5 | D | 3 |  |
| (279) Hung 2016 | 1 | L2-L5 | U | 3 |  |
| (280) Junhui 2017 | 1 | L1-L2 | S | 3 | 384^A,E,M,T^; 398^A,E,M,T^ |
| (281) Kalichman 2010 | 3 | L3-L5 | S | 3 |  |
| (282) Kim 2005 | 1 | - | U | 3 | 1137.2^A,HH^; 1321.9^A,II^ |
| (283) Kim 2013 | 1 | L1-S1 | U | 3 |  |
| (284) Koppenhaver 2018 | 2 | L4-L5 | D | 3 |  |
| (42) Kramer 2001 | 3, 5 | - | S | 3 |  |
| (285) MacDonald 2009 | 5 | L5 | S | 3 |  |
| (19) Moseley 2002 | 2, 5, 6 | L4 | D / S | 3 |  |
| (286) Skeie 2015 | 2 | - | S | 3 |  |
| (287) Sugawara 2016 | 1 | L4-S1 | S | 3 | 890^B,G,K^; 860^B,F,K^; 830^B,G,L^; 820^B,F,L^ |
| (288) Tucker 2013 | 5 | L4-L5 | D / S | 3 |  |
| (22) Ward 2009 | 5 | L1-S1 | D / S | 3 | 2390^XX^ |
| (289) Wiltse 1988 | 1 | L4 | S | 3 |  |
|  |  |  |  |  |  |
| (290) Andersen 2017 | 2 | L4-S1 | D / S | 2 |  |
| (291) Arts 2011 | 1 | L3-S1 | U | 2 | 832^A,E,T,BB^; 817^A,E,T,AA^;821^A,E,BB^; 846^A,E,AA^ |
| (292) Bae 2013 | 3 | L4 | D / S | 2 | 574.60^K,T,V,RR^; 610.30^K,V,RR^; 551.70^K,T,V,QQ^; 584.40^K,V,QQ^;557.00^K,T,V,RR^; 688.40^K,V,RR^; 538.30^K,T,V,QQ^; 673.30^K,V,QQ^ |
| (293) Bailey 2018 | 1 | L3-L4 | U | 2 | 1235.7^B,O,T^; 1158.1^B,O^ |
| (294) Chan 2012 | 2 | L4 | D | 2 | 616^B,F,K,CCC^; 716^B,F,K,DDD^; 579^B,G,K,CCC^; 707^B,G,K,DDD^; 537^A,E,F,K,CCC^; 658^A,E,F,K,DDD^;541^A,E,G,K,CCC^; 661^A,E,G,K,DDD^ |
| (295) Haig 2001 | 3 | - | U | 2 |  |
| (296) Herbert 2008 | 2 | S1 | U | 2 | 530^F,Q^; 570^F,Q^; 520^G,Q^; 540^G,Q^ |
| (297) Hebert 2014 | 1 | L4-L5 | U | 2 | 1051^E,P^ |
| (298) Hebert 2015 | 2 | L4-S1 | S | 2 |  |
| (299) Hides 1996 | 2 | L1-S1 | U | 2 | 25^C,L,GG^ |
| (300) Hides 1998 | 2, 5 | L3-S1 | D | 2 |  |
| (301) Kim 2014 | 2 | L1-L2 | U | 2 |  |
| (302) Niemelainen 2011 | 1 | L3-S1 | S | 2 | 730^B,G,O^; 690^B,F,O^; 1010^B,G,P^; 950^B,F,P^; 1110^B,G,Q^; 1080^B,F,Q^ |
| (303) Olivier 2017 | 2 | L3-L5 | U | 2 | 711^B,J^; 704^B,J^; 760^B,K^; 746^B,K^; 684^B,L^; 704^B,L^ |
| (304) Vasseljen 2006 | 5 | L4-L5 | D / S | 2 |  |
| (305) Wachi 2017 | 1, 2 | L5 | U | 2 | 791^B,G,L,LL,OO^; 760^B,F,L,LL,OO^ |
| (27) Zhao 2000 | 1, 3 | L4-S1 | U | 2 | 33.15^A,E^; 37.579^B,E^; 19.316^A,E^; 23.211^B,E^ |
| (306) Briani 2019 | 2 | L4,L5 | U | 2 |  |
| (307) Claus 2018 | 5 | L4 | D / S | 2 |  |
| (308) Cuellar 2019 | 2 | L2,S1 | U | 2 | 267 ^A,I,W, CCC,EEE^; 350 ^A,J,W, CCC,EEE^;  441.6 ^A,K,W, CCC,EEE^; 521.1 ^A,L,W, CCC,EEE^ |
| (309) Finta 2018 | 2 | L4,L5 | D | 2 |  |
| (310) Harper 2018 | 5 | - | U | 2 |  |
| (311) Kim 2018 | 2 | L4,L5 | U | 2 |  |
| (312) Masaki 2019 | 2 | L3 | U | 2 |  |
| (313) Ozcan-Eksi 2019 | 1 | L1-S1 | U | 2 |  |
| (314) Rahmani 2018 | 2 | L5-S1 | U | 2 |  |
| (315) Sarafraz 2018 | 2 | L5 | U | 2 | 51.81^A,L,X,CCC,FFF^ ; 45.54^B,L,X,CCC,FFF^ |
| (316) Shan 2018 | 4, 5 | - | S | 2 |  |
|  |  |  |  |  |  |
| (317) Akbari 2008 | 2 | L4-L5 | S | 1 |  |
| (318) Berglund 2017 | 2 | L5 | U | 1 |  |
| (319) Hosseinifar 2013 | 2 | L4-L5 | D | 1 |  |
| (320) Hosseinifar 2015 | 2 | L3-S1 | D | 1 | 8520^B,G,K^; 7899^B,F,K^ |
| (321) Ikezoe 2012 | 2 | L4 | D | 1 |  |
| (322) Joseph 2015 | 2 | L4 | D | 1 |  |
| (323) Kim 2014 | 3 | L4 | D | 1 | 386.1^A,G,K^; 386.1^A,F,K^ |
| (324) Kliziene 2015 | 2 | L4-L5 | D | 1 | 657^E,G,P^; 655^E,F,P^; 698^B,G,P^; 704^B,F,P^ |
| (50) Le Cara 2014 | 2, 1 | L4-S1 | S | 1 |  |
| (325) Masaki 2016 | 2 | L4 | D | 1 |  |
| (326) Yang 2015 | 2 | L4-L5 | U | 1 |  |
| (327) Hebert 2018 | - | - | U | 1 |  |
| (328) Nabavi 2018 | 2 | L5 | D | 1 |  |
| (329) Shahtahmassebi 2019 | 2 | L4-S1 | U | 1 |  |
|  |  |  |  |  |  |
| (52) Cho 2013 | 2 | L4 | S | 0 |  |
| (330) Hides 2011 | 2 | L5 | U | 0 | 355.0^A,E,L,X,SS,ZZ^; 401.4^B,E,L,X, SS,AA^ |
| (331) Huang 2013 | 2 | L3 | D | 0 | 588^E,J,AAA^; 700^E,J,ZZ^ |
| (332) Huang 2014 | 2 | L3 | D | 0 | 777^E,J,T,AAA^; 850^E,J,T,ZZ^ |
| (333) Huang 2014 | 2 | L3 | D | 0 | 879^B,E,J^; 761^A,E,J^ |
| (334) Huang 2014 | 2 | L5 | D | 0 | 720^A,E,L,W,AAA^; 860^A,E,L,W,ZZ^ |
| (335) Ikezoe 2015 | 2 | L4 | D | 0 |  |
| (336) Lee 2016 | 2 | - | D | 0 |  |
| (337) Noormohammadpour 2016 | 2 | L4 | D | 0 | 480^A,E,G,K^; 500^B,G,K^; 470^A,E,F,K^; 490^B,G,K^ |
| (338) Rahmani 2018 | 2 | L5-S1 | D | 0 | 124^B,G,Q^; 109^E,G,Q^; 125^B,F,Q^; 105^E,F,Q^ |
| (339) Rostami 2014 | 2 | L4 | U | 0 | 840^B,G,K,W^; 786^B,F,K,W^; 885^B,G,K,X^; 821^B,F,K,X^ |
| (340) Tsuchikane 2017 | 2 | L5 | U | 0 |  |
| (341) Hwang 2018 | - | - | U | 0 |  |
| (342) Rutkowska-Kucharska 2018 | 1 | L3,L4 | U | 0 | 210^B,F,O,W,MM,OO^; 220^B,G,O,W,MM,OO^ |
| (343) Tandon et al. 2018 | 1 | - | U | 0 | 742.67^A,W^ |

^1^ L=Location; S = Superficial; D = Deep; U=unretrievable; 1 = MRI; 2 = USI; 3 = CT; 4 = Photo; 5 = Drawing; 6 = Modelling; 7 = Stereomicroscope; 8 = Tractography.
^2^ 5 = high quality; 4 or 3 = moderate quality; ≤ 2 = low quality.
^3^ See Appendix 5
