# Supplementary material for: Inconsistent descriptions of lumbar multifidus morphology: A scoping review
Source: BMC Musculoskelet Disord. 2020 May 19;21:312. doi: 10.1186/s12891-020-03257-7 (PMC7236939; doi:10.1186/s12891-020-03257-7)
Supplement: Supplementary file 4 — Additional file 4. Studies that measured thickness of lumbar multifidus. [file 12891_2020_3257_MOESM4_ESM.docx]

**Additional file 4**

| Studies that measured thickness of lumbar multifidus, sorted by author. | | | | | | | |
| --- | --- | --- | --- | --- | --- | --- | --- |
| Author year | Method^2^ | Spine Level | L^1^ | Thickness (mm)^3^ | | | |
| (253) Aboufazeli 2019 | 2 | L4,L5 | D | 2.88 ^B,P,U,W,MM,CCC^; | | 3.205 ^A,P,U,W,MM,CCC^; | |
|  |  |  |  | 3.95 ^B,P,U,X,MM,CCC^; | | 4.465 ^A,P,U,X,MM,CCC^ | |
| (317) Akbari 2008 | 2 | L4-L5 | S | 8.6 ^A,E,P,T,U^; | 9.7 ^A,E,P,U^; | 8.8 ^A,E,P,T,U^; | 9.3 ^A,E,P,U^ |
| (168) Azadinia 2019 | 2 | L4 | D | 32.62 ^A,E,K,W,CCC,EEE^; | | 33.11 ^A,E,K,U,W,CCC,EEE^; | |
|  |  |  |  | 38.93 ^A,E,K,X,CCC,EEE^; | | 39.0 ^A,E,K,U,X,CCC,EEE^ | |
| (318) Berglund 2017 | 2 | L5 | U | 27.0 ^B,E,T,U^; | 25.8 ^B,E,T,V^; | 24.6 ^A,E,T,U^; | 23.5 ^A,E,T,V^ |
| (203) Brenner 2007 | 2 | L4-S1 | S | 26.5 ^A,E,P,W^; | 28.0 ^A,E,P,X^; | 26.5 ^A,E,Q,W^; | 27.1 ^A,E,Q,X^ |
| (52) Cho 2013 | 2 | L4 | S | 2.4 ^B,K,T,V,PP^; | 2.5 ^B,K,V,PP^ |  |  |
| (38) Creze 2019 | 5 | - | S | 5.20 ^B,PP^ |  |  |  |
| (96) Cuellar 2016 | 2 | L2-S1 | S | 26.0 ^N,W,G^; | 28.3 ­^N,X,G^; | 24.2 ^O,W,G^; | 26.6 ^O,X,G^; |
|  |  |  |  | 28.5 ^P,W,G^; | 30.2 ^P,X,G^; | 28.4 ^Q,W,G^; | 30.4 ^Q,X,G^ |
| (308) Cuellar 2019 | 2 | L2-S1 | - | 26.46 ^A,N,W, CCC,EEE^ | | 23.31^A,O,W, CCC,EEE^ | |
|  |  |  |  | 28.64 ^A,P,W, CCC,EEE^ | | 27.44^A,Q,W, CCC,EEE^ | |
| (211) Debuse 2013 | 2 | L4-L5 | D | 25.9 ^B,P,CCC^ | | | |
| (212) Dickx 2010 | 2 | L3-S1 | D | 31.0 ^B,F,O,W^; | 30.7 ^B,G,O,W^; | 34.6 ^B,F,P,W^; | 34.5 ^B,G,P,W^; |
|  |  |  |  | 35.4 ^B,F,Q,W^; | 34.2 ^B,G,Q,W^ |  |  |
| (213) Djordjevic 2014 | 2 | L4-L5 | D | 27.9 ^A,E,P,W^; | 33.9 ^A,E,P,X^; | 28.5 ^B,P,W^; | 37.6 ^B,P,X^ |
| (47) Djordjevic 2015 | 2 | L1-L5 | S | 27.0 ^E,G,W^; | 28.2 ^E,F,W^; | 34.2 ^E,G,X^; | 35.0^E,F,X^; |
|  |  |  |  | 35.7 ^E,G,Y^; | 36.8 ^E,F,Y^ |  |  |
| (309) Finta 2018 | 2 | L4,L5 | D | 24.56^A,D,G,P,W,CCC^ | | 33.49 ^A,D,G,P,X,CCC^ | |
|  |  |  |  | 23.63 ^A,D,F,P,W,CCC^ | | 33.37 ^A,D,F,P,X,CCC^ | |
| (175) Fortin 2019 | 2 | L5-S1 | D / S | 29.6 ^G,Q,W,MM,CCC^; | | 30.4 ^F,Q,W,MM,CCC^; | |
|  |  |  |  | 34.4^G,Q,W,MM,DDD^; | | 34.6 ^F,Q,W,MM,DDD^ | |
| (108) Gibbon 2017 | 2 | L4-L5 | D | 29 ^B,P,W^; | 35 ^B,P,X^ |  |  |
| (113) Hides 2014 | 2 | L4-L5 | S | 32.0 ^B,P,T^ | | | |
| (115) Hides 2016 | 2 | L2-L5 | S | 22.0 ^B,I,W^; | 24.1 ^B,I,X^; | 26.1 ^B,J,W^; | 28.0 ^B,J,X^; |
|  |  |  |  | 29.6 ^B,K,W^; | 32.6 ^B,K,X^; | 31.7 ^B,L,W^; | 34.4 ^B,L,X^ |
| (319) Hosseinifar 2013 | 2 | L4-L5 | D | 30.0 ^E,G,P,T,W^; | 30.8 ^E,F,P,T,W^; | 36.28 ^E,G,P,T,X^; | 37,06 ^E,F,P,T,X^ |
| (320) Hosseinifar 2015 | 2 | L4-S1 | D | 29.87 ^B,G,Q,W^; | 29.23 ^B,F,Q,W^; | 29.25 ^B,G,O,W^; | 29,82 ^B,F,O,W^ |
| (321) Ikezoe 2012 | 2 | L4 | D | 26.7 ^B,K,OO^; | 23.2 ^B,K,PP^; | 22.8 ^B,K,PP^ |  |
| (335) Ikezoe 2015 | 2 | L4 | D | 26.8 ^B,K,MM,PP^ | | | |
| (322) Joseph 2015 | 2 | L4 | D | 28.0 ^A,K,QQ^; | 29.0 ^A,K,RR^ |  |  |
| (127) Kiesel 2007 | 2 | L4-L5 | S | 24.8 ^B,P,W^; | 32.8 ^B,P,X^ |  |  |
| (75) Kiesel 2007 | 2 | L4-S1 | D | 12.3 ^A,E,P,EE^; | 15.4 ^A,E,P,FF^; | 11.1 ^A,E,Q,EE^; | 12.3 ^A,E,Q,OO^ |
| (301) Kim 2014 | 2 | L1-L2 | U | 35.9 ^B,M,X^ | | | |
| (311) Kim 2018 | 2 | L4,L5 | - | 23.29 ^A,E,P,T^ | 22.83 ^A,E,P,T^ |  |  |
| (74) Koppenhaver 2009 | 2 | L4-L5 | S | 34.6 ^E,P,W^; | 37.9 ^E,P,X^ |  |  |
| (53) Lariviere 2013 | 2 | L2-L5 | S | 27.6 ^E,N,X^; | 28.1 ^E,N,X^; | 35.9 ^B,N,X^; | 35.8 ^B,N,X^; |
|  |  |  |  | 32.5 ^E,P,X^; | 33.2 ^E,P,X^; | 39.6 ^B,P,X^; | 41.1 ^B,P,X^ |
| (131) Lariviere 2018 | 2 | L3-S1 | S | 30.0 ^B,G,Q^; | 29.9 ^B,F,Q^; | 28.6 ^B,G,P^; | 28.3 ^B,F,P^; |
|  |  |  |  | 26.8 ^B,G,O^; | 26.6 ^B,F,O^ |  |  |
| (336) Lee 2016 | 2 | U |  | 7.8 ^B,T,OO^; | 7.3 ^B,T,OO^ |  |  |
| (135) MacDonald 2011 | 2 | L4-S1 | D / S | 26.6 ^B,F,Q,X^; | 26.8 ^B,F,Q,X^; | 26.6 ^B,G,Q,X^; | 27.1 ^B,G,Q,X^; |
|  |  |  |  | 25.9 ^E,F,Q,X,^; | 26.3 ^E,F,Q,X^; | 26.6 ^E,G,Q,X^; | 27.3 ^E,G,Q,X^ |
| (136) Masaki 2015 | 2 | L4 | D | 23.4 ^B,K,MM,PP^ | | | |
| (325) Masaki 2016 | 2 | L4 | D | 23.3 ^B,K,MM,PP^ | | | |
| (233) Nabavi 2014 | 2 | L4-L5 | S | 26.1 ^G,P^; | 26.9 ^F,P^ |  |  |
| (235) Nuzzo 2014 | 2 | L4-L5 | S | 29.0 ^B,K,LL^; | 28.0 ^B,L, LL^; | 24.0 ^B,K,MM^ | 24.0 ^B,L,MM^ |
| (236) Partner 2014 | 2 | L4-L5 | D / S | 28.9 ^E,P,T,CCC^; | 30.4 ^E,P,T,DDD^ |  |  |
| (259) Pishnamaz 2018 | 2 | L3-L5 | S | 28.2^A,O.W,CCC,EEE^; | | 29.1 ^A,O.X,CCC,EEE^ | |
|  |  |  |  | 26.5 ^B,O.W,CCC,EEE^ | | 27.8 ^B,O.X,CCC,EEE^ | |
|  |  |  |  | 29.9 ^A,P.W,CCC,EEE^ | | 30.8 ^A,P,X,CCC,EEE^ | |
|  |  |  |  | 28.4 ^B,P.W,CCC,EEE^ | | 29.4 ^B,P.X,CCC,EEE^ | |
| (239) Seung 2007 | 3, 4 | U | D | 39.8 ^B,T^ | | | |
| (187) Shadani 2018 | 2 | L5 | - | 28.9 ^B,G,L,W,CCC^ | | 30.3 ^B,F,L,W,CCC^ | |
|  |  |  |  | 29.1 ^B,G,L,X,CCC^ | | 31.3 ^B,F,L,X,CCC^ | |
| (51) Sions 2014 | 2 | L3-S1 | S | 29.7 ^B,P,W,OO^; | 29.2 ^B,P,W,OO^; | 33.6 ^B,P,W,PP^; | 33.0 ^B,P,W,PP^ |
| (144) Sions 2015 | 2 | L4-L5 | S | 35.9 ^E,P,W^; | 40.4^E,P,X^ |  |  |
| (286) Skeie 2015 | 2 | U | S | 27.9 ^E^; | 28.9 ^E,W^; | 32.1 ^E,X^; | 29.7 ^E,X^; |
|  |  |  |  | 28.4 ^E,W^; | 30.6 ^E,W^ |  |  |
| (151) Sweeney 2014 | 2 | L4-S1 | S | 29.0 ^A,E,P,W^; | 29.9 ^B,E,P,W^; | 29.3 ^A,E,Q,W^; | 29.9 ^B,E,Q,W^; |
|  |  |  |  | 34.2 ^A,E,P,X^; | 33.7 ^B,E,P,X^; | 32.4 ^A,E,Q,X^; | 32.5 ^B,E,Q,X^ |
| (340) Tsuchikane 2017 | 2 | L5 | U | 27.3 ^B,L^; | 27.9 ^B,L^ |  |  |
| (305) Wachi 2017 | 1, 2 | L5 | U | 27.3 ^B,G,L,LL,OO^; | 26.8 ^G,F,L,LL,OO^; | 28.2 ^B,G,L,LL,OO^; | 27.9 ^G,F,L,LL,OO^ |
| (159) Wallwork 2009 | 2 | L2-L5 | S | 29.0 ^B,I,W^; | 31.0 ^B,I,X^; | 33.0 ^B,J,W^; | 34.7 ^B,J,X^; |
|  |  |  |  | 35.9 ^B,K,W^; | 37.7 ^B,K,X^; | 35.9 ^B,L,W^; | 38.1 ^B,L,X^; |
|  |  |  |  | 27.7 ^A,I,W^; | 28.9 ^A,I.X^; | 30.5 ^A,J,W^; | 31.9 ^A,J,X^; |
| (162) Wilson 2016 | 2 | L2-S1 | S | 28.0 ^B,N,W,PP^; | 28.3 ^B,N,X,PP^; | 25.4 ^B,O,W,PP^; | 25.9 ^B,O,X,PP^; |
|  |  |  |  | 32.6 ^B,P,W,PP^; | 32.8 ^B,P,X,PP^; | 29.1 ^B,Q,W,PP^; | 30.0 ^B,Q,X,PP^ |
| (163) Worsley 2012 | 2 | L3-L4 | S | 27.3 ^O,EEE^; | 27.2 ^O,FFF^ |  |  |
| (326) Yang 2015 | 2 | L4-L5 | U | 18.0 ^B,P,T^; | 20.0 ^B,P,T^ |  |  |
| (263) Yoshiko 2018 | 2 | L4,L5 | - | 29.9 ^B,P,LL,FFF^ | | 24.9^B,P,LL,FFF^ | |
| (49) Zapata 2015 | 2 | L1,L4 | S | 13.7 ^B,H,W^; | 19.7 ^B,K,W^; | 13.9 ^B,G,H,W^; | 19.6 ^B,G,K,W^; |

^1^ L=Location; S = Superficial; D = Deep; U = Unretrievable.
^2^ 1 = MRI; 2 = USI; 3 = CT; 4 = Photo; 5 = Drawing; 6 = Modelling; 7 = Stereomicroscope; 8 = Tractography.
^3^ See Appendix 5.
