# Supplementary material for: Inconsistent descriptions of lumbar multifidus morphology: A scoping review
Source: BMC Musculoskelet Disord. 2020 May 19;21:312. doi: 10.1186/s12891-020-03257-7 (PMC7236939; doi:10.1186/s12891-020-03257-7)
Supplement: Supplementary file 5 — Additional file 5. Legend of Additional files 3 and 4. [file 12891_2020_3257_MOESM5_ESM.docx]

**Additional file 5**

Legend of Appendix 3 and Appendix 4

1. Diseased side
2. Normal or healthy side
3. Group of patients with symptom duration 1-30 days
4. Group of patients with symptom duration 31-90 days
5. Group of patients with symptom duration >90 days
6. Left side
7. Right side
8. L1
9. L2
10. L3
11. L4
12. L5
13. L1-L2
14. L2-L3
15. L3-L4
16. L4-L5
17. L5-S1
18. Upper level
19. lower level
20. Baseline measurement
21. Control group (no resistive vibration exercise)
22. (Resistive vibration) exercise group
23. Resting
24. Contraction
25. Maximum contraction
26. Lean multifidus muscle
27. Conventional open approach group
28. Minimally invasive approach group
29. CSA of noncontractile components (%)
30. Expressed as a percentage of the CSA of the vertebral body
31. Positive score at multifidus lift test at L5-S1 with no weight
32. Positive score at multifidus lift test at L5-S1 with weight
33. Expressed as a percentage differences between affected and unaffected side
34. Open pedicle screw fixation group
35. Percutaneous pedicle screw fixation group
36. Expressed as a percentage by: [(thickness1-week – thicknessbaseline)/thicknessbaseline] ⁄ 100%.
37. Expressed as a percentage of differences in CSA between sides.
38. Men
39. Women
40. Degenerative Lumbar Scoliosis Group With Unilateral Radiculopathy
41. Younger adult
42. Older adult
43. Ipsilateral (to hand preference size)
44. Contralateral (to hand preference size)
45. Unilateral
46. Range between L1/L2 – L5/S1, in subjects between 10-80 years old
47. Physical activity: active (9 to 14 days)
48. Physical activity in 14 days: moderately active (1 to 8 days)
49. Physical activity in 14 days: inactive (0 days).
50. Physiological cross-sectional area calculated by: (((M(g))*(cos(θ)))/((p(g/cm3))*(Lf(cm)))
51. Unit is unknown
52. (Discectomy) no pain
53. (Discectomy) with pain
54. Fat-free muscle
55. Prone position
56. Standing position
57. Transducer Curvilinear
58. Transducer Linea
